# Salts of HCN‐Cyanide Aggregates: [CN(HCN)_2_]^−^ and [CN(HCN)_3_]^−^

**DOI:** 10.1002/anie.201915206

**Published:** 2020-04-21

**Authors:** Kevin Bläsing, Jörg Harloff, Axel Schulz, Alrik Stoffers, Philip Stoer, Alexander Villinger

**Affiliations:** ^1^ Anorganische Chemie Institut für Chemie Universität Rostock A.-Einstein-Strasse 3a 18059 Rostock Germany; ^2^ Materialdesign Leibniz-Institut für Katalyse an der Universität Rostock A.-Einstein-Strasse 29a 18059 Rostock Germany

**Keywords:** hydrogen bonding, hydrogen cyanide, solvates, structure elucidation

## Abstract

Although pure hydrogen cyanide can spontaneously polymerize or even explode, when initiated by small amounts of bases (e.g. CN^−^), the reaction of liquid HCN with [WCC]CN (WCC=weakly coordinating cation=Ph_4_P, Ph_3_PNPPh_3_=PNP) was investigated. Depending on the cation, it was possible to extract salts containing the formal dihydrogen tricyanide [CN(HCN)_2_]^−^ and trihydrogen tetracyanide ions [CN(HCN)_3_]^−^ from liquid HCN when a fast crystallization was carried out at low temperatures. X‐ray structure elucidation revealed hydrogen‐bridged linear [CN(HCN)_2_]^−^ and Y‐shaped [CN(HCN)_3_]^−^ molecular ions in the crystal. Both anions can be considered members of highly labile cyanide‐HCN solvates of the type [CN(HCN)_*n*_]^−^ (*n=*1, 2, 3 …) as well as formal polypseudohalide ions.

## Graphical abstract

**Flexible cyanides**: Bulky cations and fast crystallization allowed the isolation and structural characterization of examples of HCN‐cyanide aggregates [CN(HCN)_2_)]^−^ and [CN(HCN)_3_]^−^ in the solid state. X‐ray structure elucidation revealed hydrogen‐bridged linear [CN(HCN)_2_]^−^ and Y‐shaped [CN(HCN)_3_]^−^ molecular ions in the crystal.

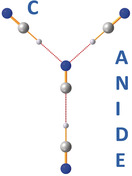

Ever since the discovery of hydrogen cyanide (HCN) by Scheele in 1782, the chemistry of HCN has been in the focus of organic, inorganic, and industrial chemistry.^1^, ^2^, ^3^, ^4^, ^5^, ^6^, ^7^, ^8^, ^9^ Scheele generated HCN by heating Prussian blue with sulfuric acid.^10^, ^11^ Modern industrial processes are based on methane and ammonia, which are reacted either with or without oxygen on a Pt contact (BMA, Andrussow process).^12^, ^13^, ^14^, ^15^ As a result of its industrial and biological^16^, ^17^, ^18^, ^19^, ^20^ importance, numerous theoretical^21^, ^22^, ^23^, ^24^, ^25^, ^26^, ^27^, ^28^ and experimental^22^, ^23^, ^29^, ^30^, ^31^, ^32^, ^33^, ^34^, ^35^, ^36^, ^37^, ^38^, ^39^, ^40^, ^41^, ^42^, ^43^, ^44^ studies on HCN and its aggregates (HCN)_*n*_ have been performed, but mostly in the gas phase (microwave rotational spectroscopy, IR or matrix IR spectroscopy). As the smallest pseudohalide,^45^ the cyanide ion, CN^−^, was the first ion to be compared with halides. It was Gay‐Lussac who prepared NC−CN from Hg(CN)_2_ in 1815 and introduced the name hydrogen cyanide by comparison with hydrogen chloride.^46^ Finally, Birckenbach and Kellermann^47^ coined molecular entities pseudohalogens when their chemistry showed strong similarities to halogens, such as the formation of an acid (HCN), a dimer (NC−CN), poorly soluble silver salts (AgCN), and disproportion in water (NC−CN+2 OH^−^→CN^−^+OCN^−^+H_2_O). This group of chemical features for a pseudohalogen^48^, ^49^ can be extended to, for example, the formation of the cationic or anionic aggregate series [H(HX)_*n*_]^+^ and [X(HX)_*n*_]^−^ (X=halogen or pseudohalogen). For example, [H(HF)_*n*_]^+^ (*n=*1, 2)^50^ and [F(HF)_*n*_]^−^ (*n=*1–4)^51^, ^52^, ^53^, ^54^ as well as the pseudohalogen analogues such as [H(HN_3_)]^+[55]^ and [N_3_(HN_3_)]^−[56]^ have been reported. More recently, salts bearing the polypseudohalides [SCN(HSCN)]^−^ have been isolated.^57^, ^58^ The first attempted synthesis of [CN(HCN)]^−^ was published by Salthouse and Waddington, who treated [*n*Pr_4_N]CN with pure HCN. This yielded an oily liquid after pumping off the excess HCN.^59^ The first experimental report on [H(HCN)_*n*_]^+^ (*n=*1–4) aggregates in the gas phase was published by Moet‐Ner, who utilized a pulsed high‐pressure mass‐spectrometric technique to determine thermochemistry data of these species.^60^ Ten years later, Moet‐Ner et al. reported thermochemical measurements and computations for HCN‐cyanide aggregates in the gas phase by utilizing the same mass‐spectrometric method.^61^ It was found that the aggregate series [CN(HCN)_*n*_)]^−^ (*n=*1–7) is formed exothermically in each HCN addition step (Δ*H*° between −20.7 and −7.6 kcal mol^−1^). The energy of the hydrogen bond in [CN(HCN)]^−^ was also determined from ion cyclotron resonance measurements of CN^−^/HCN‐exchange equilibria.^62^ The group of Fehlhammer isolated [M‐CN⋅⋅⋅H⋅⋅⋅NC‐M]^−^ complexes (M=Cr(CO)_5_) from the reaction of [Ph_4_As][Cr(CN)(CO)_5_] and [Cr(CO)_5_CNH].^63^ More recently, Riedel and co‐workers reported the isolation of pseudohalogen halides of the type [Br(BrCN)]^−^ and [Br(BrCN)_3_]^−^.^64^ As we had recently been studying the stabilization of HCN dimers by strong Lewis acids, we investigated the reaction of Lewis bases such as CN^−^ and HCN to isolate salts containing HCN‐cyanide aggregates of the type [CN(HCN)_*n*_]^−^ (*n=*1, 2, 3 …). One of the biggest problems with the use of pure polar HCN_(l)_ (*μ*=2.98 D)^65^ is its high toxicity (180–270 ppm are fatal),^66^ low boiling point (b.p.: 25.6 °C),^67^ and relatively high melting point (m.p.: −13.4 °C),^67^ which leaves only a relatively small temperature window (−12–+25 °C, 1 atm) for working in the liquid phase. In addition, there is a tendency for spontaneous polymerization or even explosion, which can be triggered by small traces of bases. Here we report the reaction of cyanide ions in pure liquid HCN and the problems that needed to be solved, which finally led to the isolation of salts with the [CN(HCN)_*n*_]^−^ ions (*n=*2, 3). Since all the reactions were carried out without further solvent, the reaction medium can be considered as a mixture with the properties of an ionic liquid.^68^

We started this project with the synthesis of pure cyanides of the type [WCC]CN ([WCC]^+^=[Et_3_NMe]^+^, [*n*Pr_3_NMe]^+^, [Ph_3_PMe]^+^),^69^ which were then treated with an excess of liquid HCN (generated from NaCN and stearic acid (m.p.: 69 °C) at 80–100 °C under vacuum).^70^, ^71^, ^72^ The addition of an excess of HCN to neat [WCC]CN at −10 °C led to the cyanide salt dissolving at once and almost instantaneous polymerization was observed with formation of a dark brown highly viscous oil. To avoid fast polymerization, we used [Ph_4_P]CN and [Ph_3_PNPPh_3_]CN (=[PNP]CN), which contain bulkier and more symmetrical cations (cf. [Et_3_NMe]^+^ 0.20, [*n*Pr_3_NMe]^+^ 0.30, [Ph_3_PMe]^+^ 0.38, [Ph_4_P]^+^ 0.46, [PNP]^+^ 0.70 nm^3^). Indeed, the reaction of [Ph_4_P]CN with liquid HCN at −10 °C led first to a suspension. After allowing this mixture to warm up to 0 °C, the immediate dissolution of the cyanide salt and a color change of the liquid phase from colorless to slight yellow were observed. Once the cyanide salt was completely dissolved, the mixture was quickly cooled to −10 °C and placed in a refrigerator for a maximum of 3 h to obtain a large number of crystals suitable for X‐ray structure elucidation. The same procedure was applied when [PNP]CN was treated with liquid HCN, which also resulted in the formation of a reasonable quantity of colorless crystals after 15 min. Fast crystallization is essential, otherwise the process of decomposition progresses too quickly. We had to try several times to obtain suitable crystals within this time limit. Interestingly, while X‐ray studies revealed unequivocally the presence of hydrogen‐bridged linear [CN(HCN)_2_]^−^ ions in the case of the [Ph_4_P]^+^ salt (**1**), Y‐shaped [CN(HCN)_3_]^−^ anions were found with [PNP]^+^ (**2**) as the counterion (Figures 1 and 2). Moreover, we also suspended [WCC]CN ([WCC]^+^=[*n*Pr_3_PMe]^+^, [Ph_4_P]^+^]) in TMS−CN and added methanol for the in situ generation of HCN. In neither case were we able to observe the formation of any HCN‐cyanide aggregates. However, for [Ph_4_P]CN we noticed the precipitation of traces of a black amorphous solid, probably as a result of the polymerization of HCN.

**Figure 1:**
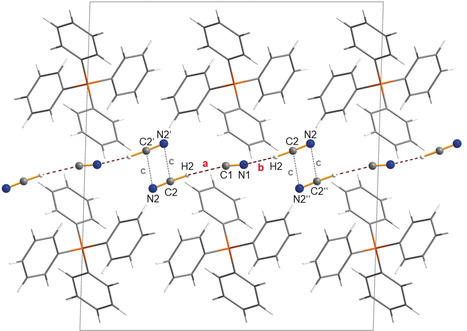
Ball‐and‐stick (anions) and wireframe (cations) representations of the molecular structure of [Ph_4_P][CN(HCN)_2_] (**1**) in the crystal. View along *[010]*. The linear molecular anion is formed by two hydrogen bridges (**a** and **b**). A chain is formed when short contacts (**c**) are considered. A section of such a chain is shown. Selected distances are given in Å, non‐hydrogen atom distance in the H‐bridges: C1−C2 3.32(1), N1−C2 2.95(1); ***a*** **=**2.27, ***b*** **=**1.90. Short interionic distances **c**: C2−N2′ 3.713(9). Disorder is not shown (see Figure S2). Only the major isomer, the thermodynamically most favored isomer, is depicted.^87^

**Figure 2:**
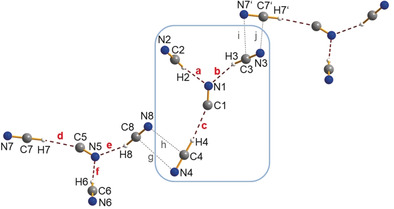
Ball‐and‐stick representation of the [CN(HCN)_3_]^−^ ion in a crystal of [PNP][CN(HCN)_3_]. The Y‐shaped molecular anion is formed by three hydrogen bridges (**a**–**c** and **d**–**f**). A chain is formed when short contacts (**g**–**j**) are considered. A section of such a chain is shown, while the cations are omitted for clarity. Selected distances are given in Å, non‐hydrogen atom distance in the H‐bridges: N1–C2 3.082(3), N1–C3 3.074(2), C1–C4 3.148(3), N5–C6 3.082(3), N5–C8 3.083(3), C5–C7 3.141(3). ***a*** **=**2.15, ***b*** **=**2.16, ***c*** **=**2.20, ***d*** **=**2.20, **e**=2.16, **f**=2.14. Short interionic distances: **i** C3–N7′ 3.477(3), **j** N3–C7′ 3.575(3), **g** C8–N4 3.504(3), **h** N8–C4 3.602(3).^87^

Both salts, [Ph_4_P][CN(HCN)_2_] (**1**) and [PNP][CN(HCN)_3_] (**2**), are very labile, that is, they decompose immediately they are removed from the HCN solution. Even in the HCN solvent at −10 °C, increasing decomposition was observed, which eventually resulted in high viscosity, black material.^73^, ^74^ Therefore, it was very difficult to obtain further analytical data. Solution NMR data (with pure HCN as solvent) at −12 °C showed a resonance in the ^13^C NMR spectrum for the solvated [CN(HCN)_*n*_]^−^ ion at −169.2 ppm, while in the ^1^H NMR spectrum only a very broad resonance was found at 4.66 ppm (half‐width: Δ*ν*_1/2_=1160 Hz; cf. 4.00 ppm with Δ*ν*_1/2_=1000 Hz for HCN in CD_2_Cl_2_; see Figures S11 and S12 in the Supporting Information). Raman data of both salts were obtained from the crystals used for the X‐ray structure elucidation as well as from the reaction mixture in an HCN (and DCN) solution. Besides experimental problems, as a result of the highly dynamic systems with respect to the hydrogen bonding and the position of the central CN^−^ ion (see below), it is very difficult to obtain reasonable spectra for the hydrogen as well as of the deuterated systems. Only very weak and broad bands could be detected for the deuterated species at 2519 ([PPh_4_]^+^) and 2504 cm^−1^ ([PNP]^+^; cf. 2575 cm^−1^ for DCN⋅⋅⋅DCN−B(C_6_F_5_)_3_)^72^ for the ν_C‐D_ stretching frequencies, which could not be further resolved.^75^, ^76^, ^77^ Broad bands for the ν_CN_ stretch appear in the expected region 2081/2057(br) ([CN(HCN)_2_]^−^) and 2073/2055(br) cm^−1^ ([CN(HCN)_3_]^−^), which are shifted to smaller wavenumbers on H/D exchange (2071/1906(br) and 2070/1906(br) cm^−1^; cf. 2097 cm^−1^ for pure HCN, 1895 cm^−1^ for pure DCN, both reference materials at 233 K).

Crystals of [Ph_4_P][CN(HCN)_2_] (**1**) and [PNP][CN(HCN)_3_] (**2**) were selected at −20 °C and −10 °C, respectively, from an open HCN solution. *Note*: Mounting of the crystals from liquid HCN has to be done under a point fume cupboard since highly toxic HCN has a rather high vapor pressure even at this temperature. **1** crystallized in the monoclinic space group *C*2/*c* with four formula units per cell. As depicted in Figure 1, the cell contains well‐separated cations and anions. Interestingly, between the linear [CN(HCN)_2_]^−^ anions there is a head‐to‐tail arrangement of the two terminal CN groups, thereby leading to a four‐membered centrosymmetric dimer (N2⋅⋅⋅C2′/C2⋅⋅⋅N2′), however with rather long distances (*d*(N2⋅⋅⋅C2′)=*d*(C2⋅⋅⋅N2′)=3.713 Å=**c**). This might be attributed to very weak van der Waals interactions resulting in the formation of {[CN(HCN)_2_]^−^}_∞_ chains along the *a*‐axis. In fact, this effect can be considered secondary, since the arrangement of the bulky cations seems to be the primary effect responsible for the size of the cavities, in which the anions are located. For comparison, structural studies of HCN also indicated that HCN forms linear hydrogen‐bonded aggregates in all its phases.^41^, ^78^, ^79^ For example, there are infinite, parallel, linear H‐bridged chains in HCN crystals.^80^ The most prominent structural feature is the topology of the nearly linear [CN(HCN)_2_]^−^ anions. As a consequence of disorder, different connectivities can be discussed: NC−H⋅⋅⋅NC⋅⋅⋅H−CN (73 %), NC−H⋅⋅⋅NC⋅⋅⋅H−NC (12 %)/NC−H⋅⋅⋅CN⋅⋅⋅H−NC (12 %), and CN−H⋅⋅⋅CN⋅⋅⋅H−NC (3 %) in accord with the computed relative thermodynamic stability of these isomers in the gas phase (see Figures 3 and 4 as well as Figure S2 in the Supporting Information; isomers **L_D_1**–**L_D_3**). In addition, all the hydrogen atoms were localized to form a molecular [CN(HCN)_2_]^−^ anion with a formal central CN^−^ ion (C1−N1), which is terminally coordinated through one CN⋅⋅⋅H−C (**a**, *d*(N1⋅⋅⋅C2)=2.95(1) Å) and one NC⋅⋅⋅H−C hydrogen bond (**b**, *d*(C1⋅⋅⋅C2)=3.32(1) Å) by one HCN molecule each. A (non‐hydrogen atom) hydrogen‐bond distance of 2.95(1) Å compared to 3.18 Å in solid HCN^80^ or 3.04(3) Å in HCN⋅⋅⋅HCN−B(C_6_F_5_)_3_ 72 indicates a bond of considerable strength. The C−H⋅⋅⋅CN hydrogen bond at 3.32(1) Å is in the range of a medium‐strength C−H⋅⋅⋅C interaction^81^ and comparable with that found in a biscarbene‐proton complex reported by Arduengo et al. (3.20(5) Å).^82^ The disolvate ion [CN(HCN)_2_]^−^ can also be regarded as a monosolvate ion (Figure 3) that is further stabilized by weak coordination of a second HCN molecule.

**Figure 3:**
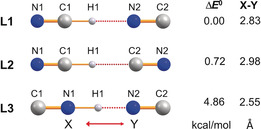
Computed isomers of the monosolvate ion [CN(HCN)]^−^ along with the relative energies (pbe0/aug‐cc‐pvtz, see Table S5).

**Figure 4:**
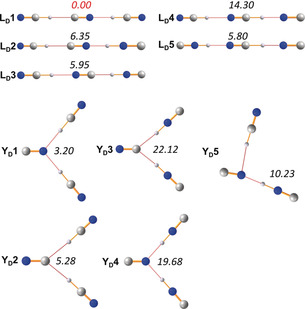
Computed isomers of the disolvate ion [CN(HCN)_3_]^−^ along with relative energies (pbe0/aug‐cc‐pvtz, in italics) in kcal mol^−1^ (see Table S6).

[PNP][CN(HCN)_3_] (**2**) crystallized in the triclinic space group *P* $\bar{1}$ with four formula units per cell. As the [PNP]^+^ cations are considerably larger than the [Ph_4_P]^+^ ions (0.70 versus 0.46 nm^3^), larger voids are formed in the crystal, now filled with larger hydrogen‐bonded, Y‐shaped [CN(HCN)_3_]^−^ ions. Again, well‐separated cation and anions are found; however, the Y‐shaped, hydrogen‐bridged trisolvate ions exhibit four very weak head‐to‐tail interactions (**i**/**j** and **g**/**h**) that lead to the formation of anion strands (Figure 2 and see Figure S4). The [CN(HCN)_3_]^−^ anion can be imagined as being formed from the disolvate ion [CN(HCN)_2_]^−^ by the addition of another HCN molecule (because of the larger void) to the central nitrogen atom of the formal CN^−^ ion. Consistent with the calculated thermodynamically most preferred isomer (see below), each trisolvate anion has three coordinated HCN molecules with three covalent C−H bonds that form three hydrogen bonds, two C−H⋅⋅⋅NC (**a**/**b** and **e**/**f**) and one C−H⋅⋅⋅CN (**c** and **d**). The non‐hydrogen atom distances in the two C−H⋅⋅⋅NC hydrogen bridges at 3.07(1) and 3.08(1) Å are a little elongated compared to the one in the [CN(HCN)_2_]^−^ ion. This can be attributed to the increase in the coordination number at the central N atom of the CN^−^ ion. Interestingly, the C−H⋅⋅⋅C distance decreased to 3.14(1) Å. In contrast to the computed gas‐phase structure, both structures show the [CN(HCN)_2_]^−^ ions in **1** and the formal [CN(HCN)_3_]^−^ units in **2** to deviate slightly from linearity, and the [CN(HCN)_3_]^−^ ions also slightly from planarity. This can be attributed to very weak energy potentials^62^ for slight changes in the angles and dihedral angles. It should be mentioned that a linear structure with an association degree of *n=*3 was also determined experimentally for the liquid HCN phase. In addition, density and heat capacity measurements of HCN gas proved the presence of (HCN)_*n*_ oligomers, especially dimeric and trimeric (HCN)_*n*_ at 25 °C and 1 atm, which were always characterized as hydrogen‐bonded linear species by means of rotational spectroscopy.^23^, ^24^, ^25^, ^34^, ^35^, ^41^, ^77^, ^78^, ^79^, ^83^

To obtain consistent structural data of all possible isomers from the isolated HCN‐cyanide aggregates in addition to the thermodynamic data, DFT (pbe0+dispersion corrections) and CCSD(T) calculations utilizing an aug‐cc‐pvtz basis set were performed (see the Supporting Information). In the following, we will only discuss the DFT results. In accord with other theoretical studies,^61^, ^62^, ^84^, ^85^, ^86^ there are three possible stable linear isomers of the monosolvate [CN(HCN)]^−^ (Figure 3). As shown in Figure 3, isomer **L1** with one covalent C−H and one H⋅⋅⋅N bridge is the thermodynamically favored isomer; however, the energy difference between **L1** and **L2** is rather small at 0.72 kcal mol^−1^. For the disolvate isomers (Figure 4), linear, *C*_∞*v*_‐symmetric isomer **L_D_1**, which can be derived from the most stable monosolvate structure **L1**, is the thermodynamically favored structure. Interestingly, all other linear structures are significantly less favored (>5.8 kcal mol^−1^). However, a nonlinear, second type of structure was found to be a stable minimum, which represents either a *C*_2*v*_‐ or *C*_s_‐symmetric, hydrogen‐bridged Y‐shaped species. The lowest energy Y‐shaped isomer (**Y_D_1**) is only 3.20 kcal mol^−1^ less favored, which becomes important in the case of the trisolvate structures, where such a Y‐shaped structure (**Y_T_1**) is now the energetically best minimum structure amongst all (found 32) isomers, in accord with our X‐ray data (Figures 4 and 5). The lowest energy linear structure (**L_T_3**), however, is only 1.39 kcal mol^−1^ less favored. Furthermore, a new, nonplanar type of structure (**C**) was found which is only 3.65 kcal mol^−1^ less favored (**C1**). Two things can be generalized from these thermodynamic considerations: i) The energetically preferred isomer is always the one with the maximum number of covalent NC−H bonds, the largest number of C−H⋅⋅⋅N hydrogen bonds, and the lowest number of isonitrile groups. ii) All favored isomers are those that were also found in the X‐ray structures. With respect to the non‐hydrogen atom distance of the hydrogen bond, the N−H⋅⋅⋅N hydrogen bonds are usually shorter (*d*(N⋅⋅⋅N)=2.53–2.76 Å) than the C−H⋅⋅⋅N hydrogen bonds (*d*(C⋅⋅⋅N)=2.72–3.14 Å), while the C−H⋅⋅⋅C distances are usually the longest (*d*(C⋅⋅⋅C)=2.90–3.20 Å).

**Figure 5:**
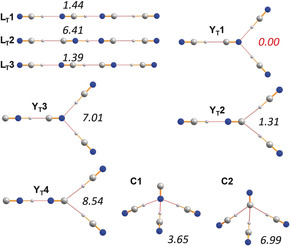
A selection of computed isomers of the trisolvate ion [CN(HCN)_3_]^−^ along with the relative energies (pbe0/aug‐cc‐pvtz, in italics) in kcal mol^−1^. Only those isomers are listed which contain exclusively H−CN bonds, as they always represent the energetically favored isomers (see Table S8).

Meot‐Ner et al.^61^ had already indicated by ab initio computations on [CN(HCN)_*n*_]^−^ ions that very flat potentials can be assumed for slight changes in the geometry, such as angles and dihedral angles, as well as for the migration of a proton. We had a closer look at the rotation potential of the central CN^−^ ion in a flexible (HCN)_*n*_ framework (*n=*2, 3; see Figures S14 and S15). These computations show that a 360° rotation is associated by an activation barrier of 7.0 (*n=*2) and 3.7 kcal mol^−1^ (*n=*3); that is, the central CN^−^ ion in [CN(HCN)_*n*_]^−^ ions may rotate almost freely in these aggregates at ambient temperatures. This suggests a nearly spherical character of the anion within these complexes, which might be one of the major reasons for the observed disorder in the solid‐state structure of [Ph_4_P][CN(HCN)_2_]. The stepwise formation of the HCN solvates [CN(HCN)_*n*_]^−^ were computed to be exothermic and exergonic for *n=*1–3 (Table S10); however, the larger the value of *n*, the less negative are the Δ_*n*_ *H*°_298_/Δ_*n*_ *G*°_298_ values (Δ_*n*_ *H*°_298_: −23.6, −18.7, and −13.0; Δ_*n*_ *G*°_298_: −15.9, −11.1, and −7.0 kcal mol^−1^; cf. from mass spectrometry: Δ_1_ *H*°_298_=−20.1±1.6,^84^ and −20.7,^61^ as well as Δ_2_ *H*°_298_=−16.4, and Δ_3_ *H*°_298_=−12.6 kcal mol^−1^).^61^

In conclusion, we have isolated and structurally characterized the first examples of HCN‐cyanide aggregates, [CN(HCN)_*n*_]^−^ (*n=*2, 3), in the solid state. As a consequence of the hydrogen bonding within the anions of the title compounds, these may be regarded as fragments of the solid structure of HCN upon (partial) deprotonation. In accord with computations, linear [CN(HCN)_2_]^−^ can be regarded as a cyanide ion stabilized by two hydrogen‐bonded HCN molecules, while Y‐shaped [CN(HCN)_3_]^−^ is energetically slightly preferred over an analogous linear isomer. There is always only a small change in the stability when CN^−^ and HCN do not form a linear complex, thus indicating a highly dynamic system at ambient temperatures, as proven by computed rotational potentials within either a linear or a Y‐shaped HCN framework. With this study we want to close a gap in HCN main group pseudohalogen chemistry that began with the discovery of HCN over two centuries ago.

## Experimental Section

**Caution**! HCN is highly toxic and can decompose explosively under various conditions! Appropriate safety precautions (HCN detector, gas mask, low temperatures) should be taken.

## Conflict of interest

The authors declare no conflict of interest.

## Supporting information

As a service to our authors and readers, this journal provides supporting information supplied by the authors. Such materials are peer reviewed and may be re‐organized for online delivery, but are not copy‐edited or typeset. Technical support issues arising from supporting information (other than missing files) should be addressed to the authors.

Supplementary
