# Supplementary material for: Salts of HCN‐Cyanide Aggregates: [CN(HCN)2]− and [CN(HCN)3]−
Source: Angew Chem Int Ed Engl. 2020 Apr 21;59(26):10508–13. doi: 10.1002/anie.201915206 (PMC7317722; doi:10.1002/anie.201915206)
Supplement: Supplementary file 1 — Supplementary [file ANIE-59-10508-s001.pdf]

Angewandte Chemie
Eine Zeitschrift der Gesellschaft Deutscher Chemiker

Supporting Information

Salts of HCN-Cyanide Aggregates: $[CN(HCN)_2]^-$ and $[CN(HCN)_3]^-$

Kevin Bläsing, Jörg Harloff, Axel Schulz, Alrik Stoffers, Philip Stoer, and Alexander Villinger*

anie_201915206_sm_miscellaneous_information.pdf

Supporting Information

Salts of HCN-Cyanide-Aggregates : $[CN(HCN)_n]^-$ (n = 2 - 3)

Kevin Bläsing, Jörg Harloff, Axel Schulz, Philip Stoer, Alrik Stoffers and Alexander Villinger*

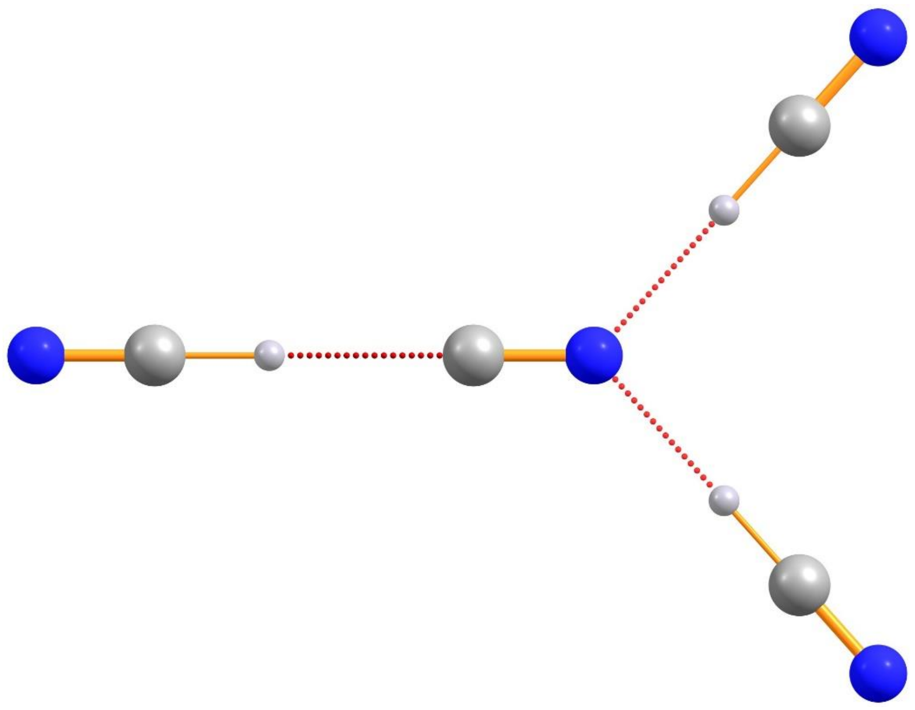

Content

1. Experimental Details 2
2. Structure Elucidation 4
3. Experimental Description 10
4. Computational Details 27
5. References 51

1. Experimental Details

General: All manipulations were carried out in oxygen- and moisture-free conditions in an argon atmosphere using standard Schlenk or dry-box techniques if not mentioned otherwise. Acetonitrile (acetonirile, acetonitrile-d_3) and dmso-d_6 was dried with CaH_2 and freshly distilled prior to use and stored over molecular sieves (3 Å). Acetone was dried with CaH_2, freshly distilled and stored over molar sieves (3 Å). Before using, the acetone was condensed into a new Schlenk flask. MeOH and diethyl ether were dried with Na and freshly distilled prior to use. TMS–CN (98 %, abcr) was distilled prior to use. NaCN (Merck, 95 %), KCN (Merck, 95 %), [PPh_4P]Cl (Alfa Aesar, 98 %) and [PPN]Cl (abcr, 97 %) were used as received after drying *in vacuo*. [Et_3NMe][CO_3Me], [Pr_3NMe][CO_3Me] and [Ph_3PMe][CO_3Me] were synthesized according to literature known procedures.[1] HCN and DCN were synthesized according to literature known processes.[2]

NMR Spectroscopy: ^{1}H, $^{13}C\{^{1}H\}$, $^{31}P\{^{1}H\}$ and $^{14}N\{^{1}H\}$ NMR spectra were recorded with a Bruker AVANCE 250 or a Bruker AVANCE 300 spectrometer. The chemical shifts were referenced internally to the deuterated solvent ($^{13}C\{^{1}H\}$: CD_3CN δ_{ref} = 1.3 ppm, $(CD_3)_2SO$ δ_{ref} = 39.5 ppm), to protic impurities in the deuterated solvent (^{1}H: CHD_2CN δ_{ref} = 1.94 ppm, $(CHD_2)(CD_3)SO$ δ_{ref} = 2.50 ppm) or externally ($^{31}P\{^{1}H\}$: 85 % H_3PO_4 δ_{ref} = 0.0 ppm, $^{14}N\{^{1}H\}$: CH_3NO_2 δ_{ref} = 0.0 ppm). All measurements were carried at room temperature unless otherwise denoted.[3]

IR Spectroscopy: A Bruker Alpha FT-IR spectrometer with ATR device was used.

Raman Spectroscopy: A LabRAM HR 800 Horiba Jobin YVON equipped with a Olympus BX41 microscope with variable lenses were used. A red laser (633 nm, 17mW, HeNe-laser) or a green laser (532 nm, 50 mW, air cooled, doubled frequency Nd:YAG solid state laser) were used. All measurements were carried out at ambient temperature except for hydrogen cyanide and the cyanide products. For these crystalline samples a Linkam THMS600 Temperature Controlled stage was used to cool the substances to temperatures, which are denoted in the experimental descriptions. Furthermore, hydrogen cyanide and deuterium cyanide were measured in an NMR tube as solids at −40 °C using a self-constructed NMR tube holder for the Linkam THMS600 Temperature Controlled stage.

CHN Analyses: Analysator vario micro cube from Elementar was used.

Melting points are uncorrected (*EZ*)-Melt, Stanford Research Systems. Heating rate 5 $K \cdot min^{-1}$ (clearing-points are reported).

2. Structure Elucidation

X-Ray Structure Determination: X-Ray quality crystals were selected in Fomblin YR-1800 perfluoroether (Alfa Aesar) for low-temperature applications. Single crystals were measured on a Bruker D8 Quest diffractometer using graphite-monochromated Mo Kα radiation (λ= 0.71073). The structures were solved by direct methods (*SHELXS-2014*)[6] and refined by full-matrix least squares procedures (*SHELXL-2014*).[7] Semi empirical absorption corrections were applied (SADABS/TWINABS).[8] All non-hydrogen atoms were refined anisotropically and hydrogen atoms were included in the refinement at calculated positions using a riding model.

The HCN molecule in structure **1** was found to be disordered, and was split in two parts. The occupancy of each part was refined freely (0.856(17) / 0.144(17)).

	$[PPh_4][CN(HCN)_2]$ (**1**)
formula	$C_{27}H_{22}N_3P$
M [g mol^{-1}]	419.44
color	colourless
system	monoclinic
space group	*C2/c*
a [Å]	16.8399(8)
b [Å]	7.2662(4)
c [Å]	18.8510(9)
α [°]	90
β [°]	91.771(2)
γ [°]	90
V [Å^3]	308.45(8)
Z	4
$\rho_{calc.}$ [g cm^{-3}]	1.208
μ [mm^{-1}]	0.14
$\lambda_{MoK\alpha}$ [Å]	0.71073
T [K]	123
collected reflexes	29428
independent reflexes	4323
refelxes with $I > 2\sigma(I)$	3600
$R_{int.}$	0.044
F(000)	880
R_1 (R [$F^2 > 2\sigma(F^2)$])	0.040
wR_2 (F^2)	0.107
GooF	1.05
parameter	173
CCDC #	1966285

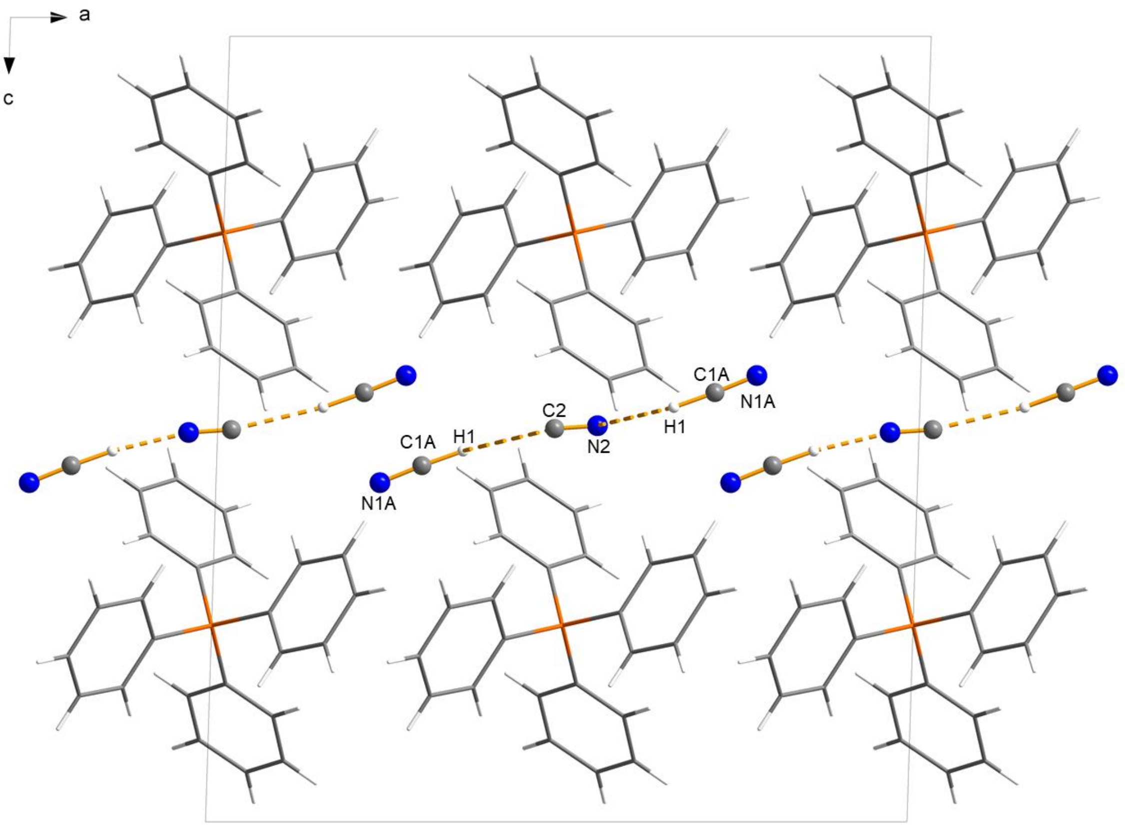

Figure S1. View of the molecular structure of [PPh_4][CN(HCN)$_2$] in the crystal along the b-axis. The cation is shown as wires/sticks model for clarity. Cyanide anions and HCN moieties forming a linear chain and are shown as ball and stick model.

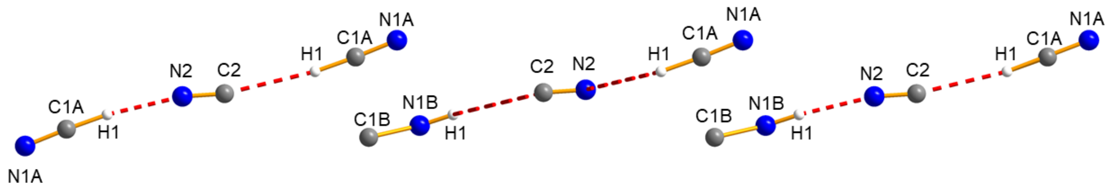

Figure S2. Three different isomers can be considered due to partial disorder of a HCN moiety and due to symmetry reasons of the central cyanide anion. Only the three isomers with the highest abundancy are shown: [NCH–NC–HCN]$^-$ (73 %, left), [CNH–CN–HCN]$^-$ (12 %, middle) and [CNH–NC–HCN]$^-$ (12 %, right).The [CNH–CN–HNC]$^-$ (3 %) anion is not shown.

	$[N(PPh_3)_2][CN(HCN)_3]$ (**2**)
formula	$C_{40}H_{33}N_5P_2$
M [g mol^{-1}]	645.65
color	colourless
system	triclinic
space group	$P\bar{1}$
a [Å]	11.3973(7)
b [Å]	15.1869(9)
c [Å]	22.821(1)
α [°]	71.898(2)
β [°]	88.024(3)
γ [°]	71.841(2)
V [Å^3]	3559.1(4)
Z	4
$\rho_{calc.}$ [g cm^{-3}]	1.205
μ [mm^{-1}]	0.16
$\lambda_{MoK\alpha}$ [Å]	0.71073
T [K]	123
collected reflexes	206951
independent reflexes	26843
refelxes with $I > 2\sigma(I)$	16739
$R_{int.}$	0.046
F(000)	1352
R_1 (R [$F^2 > 2\sigma(F^2)$])	0.049
wR_2 (F^2)	0.144
GooF	1.03
parameter	847
CCDC #	1966284

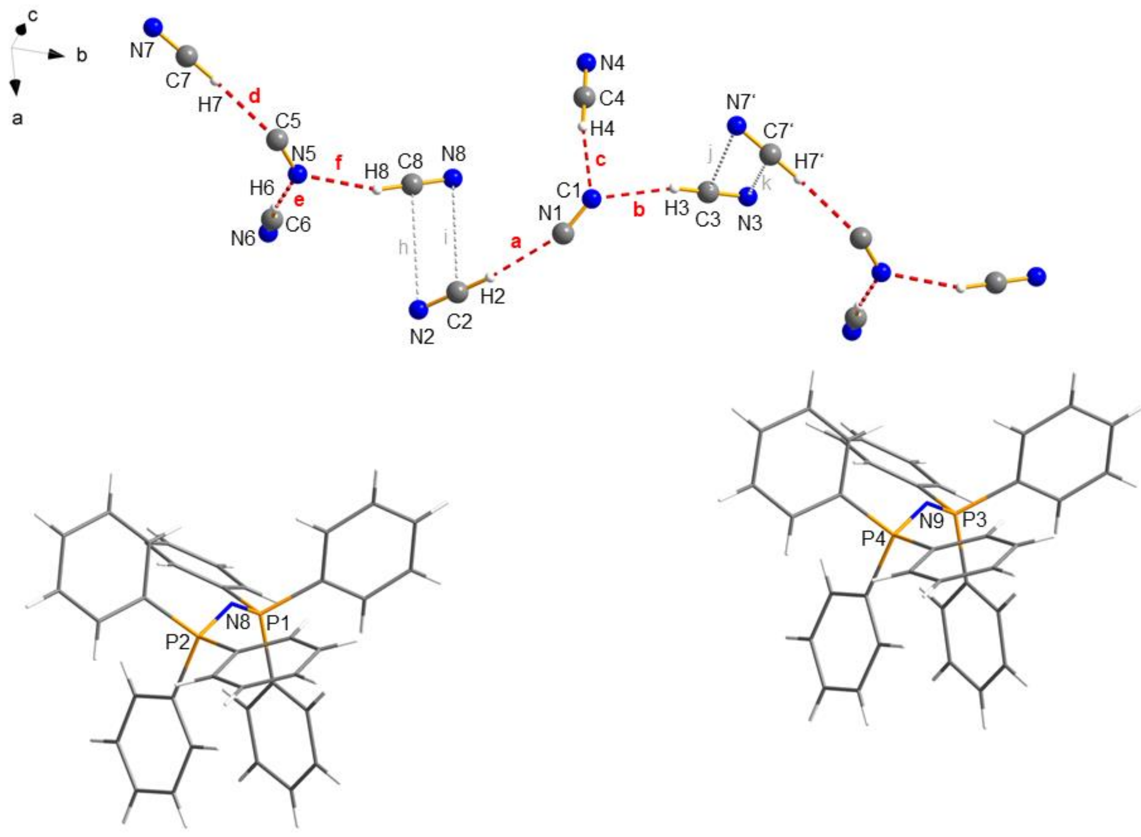

Figure S 3. Molecular structure of [PNP][CN(HCN)$_3$]. The anions are shown as ball and stick model, whereas the [PNP]$^+$ ions are shown as wires/sticks model for reasons of clarity. Dashed red lines (**a** - **f**) show hydrogen bridges of the HCN moieties and the central cyanide anion. Weak Van-der-Waals interactions (**h** - **k**) are shown as dashed grey lines, forming infinite [CN(HCN)$_3$]$^-$ chains.

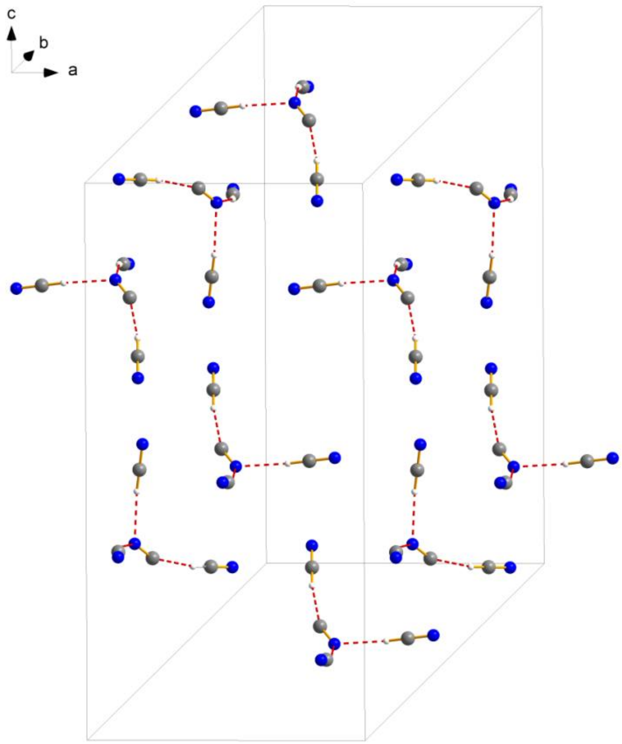

Figure S4. Ball and stick model of the anion layer in the crystal lattice, aligned in the cavities built by the [PNP]$^+$ ions. Due to reasons of clarity, the [PNP]$^+$ cation is not shown. Each cyanide anion is surrendered by three HCN moieties, forming a “Y”-shaped motive.

3. Experimental Description

Synthesis of the starting materials

The cyanide-containing starting materials [R_3EMe]CN (R = ethyl, n-propyl or phenyl; E = N or P),[4,5] *as well as, [PPh_4]CN*[6] *and [PPN]CN*[7] *were synthesized according to literature known processes.*

Synthesis of triethylmethylammonium- and tri-n-propylmethylammonium cyanide

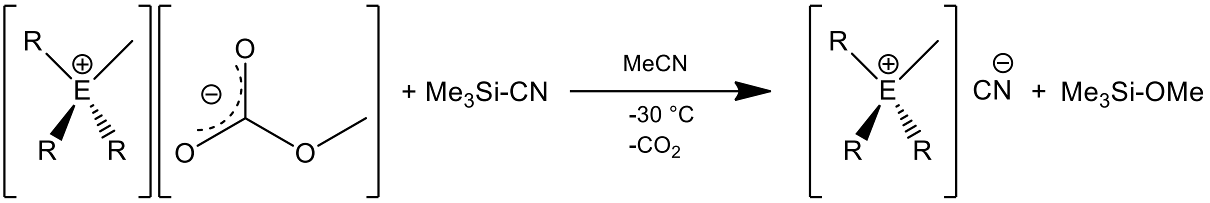

R = Ethyl, *n*-Propyl, Phenyl; E = N or P

In a three-neck round-bottom flask equipped with a stir bar, one equivalent of methyltrialkylammonium carbonate or methyltriphenylphosphonium carbonate was dissolved in acetonitrile. 1.2 eq. TMS–CN was added via syringe over a period of 20 min to the carbonate solution at −30 °C. The mixture was stirred for 30 minutes, warmed up to ambient temperature and allowed to stir for additional 30 minutes. After removing all volatiles *in vacuo*, the resulting residue was treated three times with diethyl ether. Adding and removing the diethyl ether support the elimination of left acetonitrile. Finally, the solid was dried in fine vacuum.

Triethylmethylammonium cyanide can be isolated as a colourless solid with a yield of 91 %.

$C_8H_{18}N_2$ (142.24 g/mol): **dec**. = 234 °C. **EA** found (calc.) %: C 67.44 (67.55); H 13.01 (12.75); N 19.59 (19.69). **^{1}H NMR** (298 K, CD_3CN, 300.13 MHz) δ = 3.29 (q, 6H, NC*H2,* $^3J(^1H–^1H)$ = 7 Hz); 2.90 (s, 3H, NC*H3*); 1.22 (tt, 9H, NCH_2CH_3, $^3J(^1H–^{14}N)$ = 2 Hz, $^3J(^1H–^1H)$ = 7 Hz). **^{13}C{^{1}H} NMR** (297 K, CD_3CN, 75.5 MHz) δ = 167.2 (s, *C*N$^-$); 56.5 (t, N*C*H2, $^1J(^{13}C–^{14}N)$ = 3 Hz); 47.2 (t, N*C*H3, $^1J(^{13}C–^{14}N)$ = 4 Hz); 8.0 (s, *C*H_3CH_2N). **^{14}N{^{1}H} NMR** (300 K, $(CD_3)_2SO$, 36.1 MHz) δ = −89.2 (br, (C*N*)$^-$, $\Delta\nu_{1/2}$ = 85 Hz); −321.1 (s, 1N, [Et_3*N*Me]$^+$). **IR (**25 °C, ATR, 8 scans, cm^{-1}) 787 (m), 816 (w), 874 (s), 960 (s), 1003

(vw), 1047 (s), 1082 (s), 1128 (s), 1159 (s), 1192 (m), 1221 (s), 1246 (s), 1313 (s), 1358 (s), 1398 (m), 1448 (w), 1493 (w), 2052 (s), 2951 (s), 2985 (m), 3010 (s). **Raman** (laser: 633 nm, accumulation time: 20 s, 20 scans, 297 K, cm^{-1}) 386 (3), 413 (4), 429 (3), 452 (3), 500 (3), 542 (3), 686 (7), 787 (3), 819 (3), 882 (4), 890 (4), 962 (4), 1005 (4), 1077 (4), 1129 (4), 1194 (4), 1223 (4), 1306 (4), 1348 (4), 1407 (4), 1469 (5), 1488 (4), 2053 (10), 2752 (2), 2770 (2), 2862 (2), 2896 (3), 2946 (5), 2988 (4), 3017 (2).

Tri-*n*-propylmethylammonium cyanide can be isolated as a colorless solid with a yield of 95 %.

$C_{11}H_{24}N_2$ (184.31 g/mol): **dec**. = 216 °C. **EA** found (calc.) %: C 71.69 (71.68); H 12.21 (13.12); N 15.22 (15.20). **^{1}H NMR** (300 K, $(CD_3)_2SO$, 250.13 MHz) δ = 3.21–3.12 (m, 6H, NCH_2); 2.96 (s, 3H, NCH_3); 1.64 (sex, 6H, NCH_2CH_2, $^3J(^1H–^1H)$ = 7 Hz); 0.89 (t, 9H, CH_2CH_3, $^3J(^1H–^1H)$ = 7 Hz). **$^{13}C\{^1H\}$ NMR** (300, $(CD_3)_2SO$, 62.9 MHz) δ = 166.7 (s, *C*N); 62.0 (t, NCH_2 $^1J(^{13}C–^{14}N)$ = 2 Hz); 47.5 (t, NCH_3, $^1J(^{13}C–^{14}N)$ = 4 Hz); 15.1 (s, NCH_2CH_2); 10.5 (s, CH_2CH_3). **$^{14}N\{^1H\}$ NMR** (300 K, $(CD_3)_2SO$, 18.1 MHz) δ = –319.5 (s, $[^nPr_3NMe]^+$); −89.8 (s, CN^-). **IR** (25°C, ATR, 32 scans, cm^{-1}) 3361 (VW), 3021 (W), 2969 (S), 2941 (M), 2879 (M), 2734 (VW), 2050 (VW), 1636 (VW), 1492 (S), 1455 (S), 1403 (W), 1379 (W), 1337 (W), 1284 (VW), 1214 (VW), 1183 (VW), 1039 (M), 1001 (M), 958 (VS), 915 (VW), 894 (W), 880 (W), 865 (W), 752 (S), 583 (VW), 519 (VW). **Raman** (laser: 633 nm, accumulation time: 12 s, 15 scans, 298 K, cm^{-1}) 86 (7), 322 (8), 380 (1), 474 (2), 582 (1), 751 (1), 773 (2), 865 (1), 893 (1), 914 (1), 932 (1), 1001 (1), 1035 (1), 1101 (2), 1140 (1), 1205 (1), 1319 (1), 1336 (1), 1363 (1), 1451 (3), 2051 (10), 2732 (1), 2877 (4), 2944 (6), 2979 (4), 3020 (2).

Triphenylmethylphosphonium cyanide can be isolated as a colourless solid with a yield of 84 %.

$C_{20}H_{18}NP$ (303.34 g/mol): **mp**. = 196 °C. **EA** found (calc.), %: C 79.34 (79.19), H 5.95 (5.98), N 4.54 (4.62). **^{1}H NMR** (297 K, CD_3CN, 300.13 MHz) δ = 7.91 – 7.64 (m, 15H, $P(C_6H_5)_3$); 2.93 (d, 3H, CH_3, $^2J(^1H–^{31}P)$ = 14 Hz). **$^{13}C\{^1H\}$ NMR** (297 K, CD_3CN, 75.5 MHz) δ = 167.2 (s, CN^-); 135.9 (d, *p-C*, $^4J(^{13}C–^{31}P)$ = 3 Hz); 134.3 (d, *m-C*, $^3J(^{13}C–^{31}P)$ = 11 Hz); 131.0 (d, *o-C*, $^2J(^{13}C–^{31}P)$ = 13 Hz); 120.5 (d, *i-C*, $^1J(^{13}C–^{31}P)$ = 89 Hz); 9.5 (d, CH_3, $^1J(^{13}C–^{31}P)$ = 58 Hz). **$^{31}P\{^1H\}$ NMR** (297 K, CD_3CN, 121.5 MHz) δ = 21.9 (s,

[$Ph_3PMe]^+$). **IR (**ATR, 8 scans, 298 K, cm^{-1}) 424 (m), 447 (m), 488 (s), 501 (vs), 544 (vw), 614 (w), 686 (s), 717 (s), 748 (s), 793 (w), 853 (w), 913 (s), 927 (m), 997 (w), 1030 (vw), 1074 (w), 1115 (s), 1166 (vw), 1191 (w), 1327 (w), 1341 (w), 1414 (w), 1438 (m), 1486 (w), 1587 (w), 1616 (vw), 1677 (vw), 1712 (vw), 1785 (vw), 1836 (vw), 1855 (vw), 1902 (vw), 1927 (vw), 2050 (w), 2613 (vw), 2693 (w), 2802 (w), 2887 (w), 2938 (w), 2978 (w), 2994 (w), 3011 (w), 3039 (w), 3056 (w), 3077 (w), 3091 (w), 3161 (vw). **Raman** (laser: 633 nm, accumulation time: 8 s, 25 scans, 298 K, cm^{-1}) 100 (10), 173 (1), 192 (2), 244 (2), 261 (3), 287 (1), 383 (1), 399 (1), 407 (1), 428 (1), 451 (1), 489 (1), 506 (1), 513 (1), 617 (2), 678 (3), 700 (1), 719 (1), 725 (1), 758 (1), 761 (1), 773 (1), 796 (1), 849 (1), 856 (1), 869 (1), 915 (1), 930 (1), 950 (1), 964 (1), 1002 (8), 1015 (1), 1032 (4), 1077 (1), 1105 (1), 1116 (2), 1168 (1), 1174 (1), 1195 (1), 1200 (1), 1286 (1), 1331 (1), 1341 (1), 1400 (1), 1413 (1), 1446 (1), 1489 (1), 1579 (1), 1591 (5), 2056 (3), 2566 (1), 2615 (1), 2807 (1), 2843 (1), 2892 (1), 2946 (1), 2979 (1), 2996 (1), 3014 (1), 3054 (3), 3060 (5), 3082 (1), 3145 (1), 3153 (1), 3177 (1).

Synthesis of μ-nitridobis(triphenylphosphane) cyanide

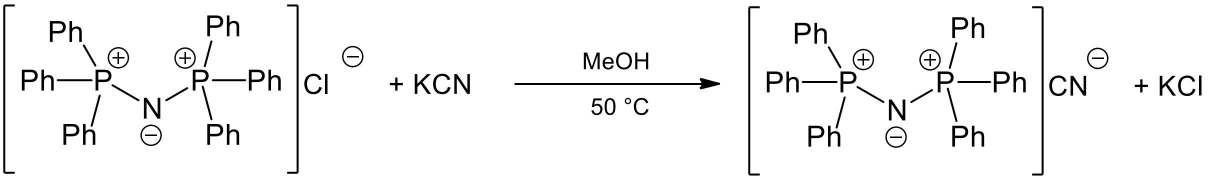

[PPN]Cl (2.54 g, 4.42 mmol) was placed in a Schlenk flask along with KCN (0.84 g, 13.27 mmol, 3 eq.). MeOH (15 mL) was added which resulted in a suspension. The mixture was warmed up to 50 °C and stirred for 10 minutes. The suspension was cooled to 0 °C with ice for additional 30 minutes and transferred by filtration to a new suspension of KCN (0.15 g, 2.31 mmol) and MeOH (5 mL). The resulting suspension was stirred for 5 minutes at room temperature and reduced in volume (to 10 mL). The mixture was placed in a refrigerator overnight. The reaction mixture was filtered and all volatiles were removed *in vacuo*. The solid was dried for 15 minutes at elevated temperatures (50 °C) for 20 minutes *in vacuo*. Acetone (10 mL) was added leading to nearly complete dissolution of the solid. The mixture was cooled to 0 °C with ice for 15 minutes and was filtered again. Adding diethyl ether (50 mL) led to formation of a

colorless precipitate which was filtered-off and dried in vacuo for three hours at 50 °C. [PPN]CN could be obtained as colourless solid (1.5 g, 2.65 mmol) in yields of 60 %.

$C_{37}H_{30}N_2P_2$ (564.60 g/mol): **dec**. = 230 °C. **EA** found (calc.), %: C 78.31 (78.71), H 5.49 (5.36), N 4.40 (4.96). **^{1}H NMR** (297 K, CD_3CN, 300.13 MHz): δ = 7.70 - 7.44 (m, 30 H, Ph). **^{13}C{^{1}H} NMR** (297 K, CD_3CN, 62.9 MHz): δ = 128.3 (dd, *i*-C, $^1J(^{13}C–^{31}P)$ = 108 Hz, $^1J(^{13}C–^{31}P)$ = 3 Hz); 130.5 (m, *o*-C); 133.3 (m, *m*-C); 134.7 (s, *p*-C); *n.o.* (CN^-). **^{31}P{^{1}H} NMR** (297 K, CD_3CN, 121.5 MHz): δ = 21.16 (s, $[PPN]^+$). **IR** (ATR, 16 scans, 298 K, cm^{-1}): $\tilde{v}$ = 437 (m), 492 (vw), 530 (vw), 546 (w), 616 (s), 688 (w), 719 (w), 748 (m), 764 (s), 800 (s), 849 (vs), 927 (s), 995 (s), 1024 (s), 1039 (s), 1078 (s), 1111 (w), 1160 (s), 1181 (s), 1261 (m), 1284 (m), 1323 (s), 1434 (m), 1482 (s), 1587 (s), 1628 (vs), 1671 (vs), 1679 (vs), 1708 (vs), 1776 (vs), 1826 (vs), 1902 (vs), 1980 (vs), 2057 (vs), 2798 (vs), 2988 (s), 3019 (s), 3050 (s). **Raman** (laser: 633 nm, accumulation time: 4 s, 15 scans, 298 K, cm^{-1}): $\tilde{v}$ = 173 (1), 186 (1), 199 (1), 231 (1), 242 (1), 250 (2), 269 (1), 283 (1), 308 (1), 328 (1), 354 (1), 399 (1), 491 (1), 527 (1), 547 (1), 615 (2), 664 (2), 702 (1), 726 (1), 746 (1), 806 (1), 857 (1), 890 (1), 927 (1), 939 (1), 1002 (7), 1026 (3), 1072 (1), 1111 (2), 1160 (1), 1184 (1), 1234 (1), 1279 (1), 1313 (1), 1332 (1), 1383 (1), 1437 (1), 1481 (1), 1575 (1), 1588 (2), 1847 (1), 2056 (1), 2086 (1), 2814 (1), 2893 (1), 2959 (1), 2992 (1), 3010 (1), 3028 (1), 3057 (2), 3068 (1), 3148 (1), 3175 (1).

Synthesis of tetraphenylphosphonium cyanide

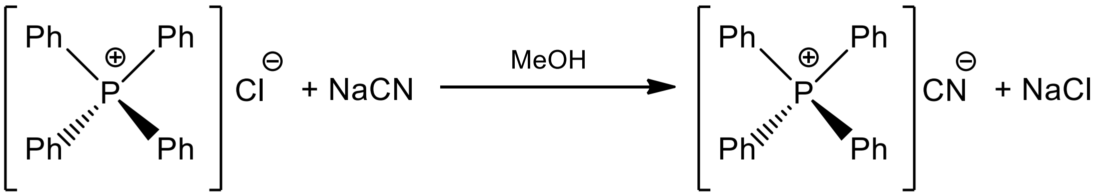

$[PPh_4]Cl$ (3.40 g, 9.08 mmol) was placed in a Schlenk flask along with NaCN (1.34 g, 27.30 mmol, 3 eq.). MeOH (11 mL) was added which resulted in a suspension. The mixture was stirred for 24 hours at room temperature and the solvent was removed in vacuo. After shortly drying the remaining solid, the residue was suspended in 9 mL of acetonitrile. The suspension was filtered and the solvent was removed in vacuo. Crystals could be obtained by recrystallization from a benzene and acetonitrile mixture, leading to slightly pink crystals (0.50 g, 1.37 mmol) in yields of 15 %.

$C_{25}H_{20}NP$ (365.41 g/mol): **dec**. = 220 °C. **EA** found (calc.), %: C 81.06 (82.17), H 5.73 (5.52), N 3.86 (3.83). **^{1}H NMR** (297 K, CD_3CN, 300.13 MHz): δ = 7.72 (m, 16 H, *o*-Ph, *m*-Ph); 7.92 (m, 4 H, *p*-Ph). **^{13}C{^{1}H} NMR** (297 K, CD_3CN, 125.8 MHz): δ = 119.1 (d, *i*-C, $^1J(^{13}C–^{31}P)$ = 88 Hz); 131.4 (d, *o*-C, $^2J(^{13}C–^{31}P)$ = 13 Hz); 135.8 (d, 8 *m*-C, $^3J(^{13}C–^{31}P)$ = 10 Hz); 136.5 (d, *p*-C, $^4J(^{13}C–^{31}P)$ = 3 Hz); 165.9 (br, $CN^–$). **^{31}P{^{1}H} NMR** (297 K, CD_3CN, 121.5 MHz): δ = 22.91 (s, $[PPh_4]^+$). **IR** (ATR, 32 scans, 298 K, $cm^{–1}$): $\tilde{v}$ = 3054 (w, br), 2047 (vw), 1584 (m), 1481 (m), 1434 (s), 1340 (vw), 1313 (w), 1277 (vw), 1182 (w), 1159 (vw), 1104 (vs), 1027 (w), 995 (m), 937 (vw), 857 (w), 764 (m), 719 (vs), 688 (vs), 615 (w). **Raman** (laser: 633 nm, accumulation time: 30 s, 4 scans, 298 K, $cm^{–1}$): $\tilde{v}$ = 95 (10), 115 (9), 198 (3), 211 (2), 257 (6), 281 (2), 362 (2), 418 (2), 441 (2), 460 (2), 503 (2), 535 (2), 562 (2), 618 (3), 640 (2), 679 (4), 698 (2), 726 (3), 779 (2), 796 (2), 863 (3), 935 (3), 973 (3), 986 (3), 1002 (9), 1029 (5), 1075 (3), 1098 (5), 1106 (3), 1136 (3), 1160 (3), 1181 (3), 1232 (3), 1329 (3), 1392 (3), 1436 (3), 1476 (3), 1573 (3), 1585 (5), 1589 (5), 1724 (2), 1833 (2), 2050 (3), 2950 (1), 2994 (1), 3047 (3), 3060 (3), 3144 (1), 3166 (1), 3203 (1).

General information about the synthesis of [WCC][(CN)(HCN)$_x$]$^-$ (x = 2, 3)

All compounds were found to be very labile. In all experiments, a color change to yellowish/brownish could be observed, as soon as the cyanide containing salt dissolves in HCN. The mixture starts to polymerize to black oil when standing at room temperature for a few minutes. Even if a freshly prepared mixture is placed in the refrigerator at −20 °C, highly viscous oil is obtained overnight. In both cases, crystals of the products were separated by crystal picking from the reaction mixture at low temperatures and immediately placed at a low-temperature stage with inert atmosphere (N_2-stream) for X-ray or Raman analysis. Because the crystals of both materials were highly labile regarding to moisture and temperature (liquefying to brownish, oily polymer), no analytical data for IR, EA, MS or NMR could be obtained from the pure crystals. The product yields could not be determined since the crystals could not be separated completely out of the brown oil and due to decomposition of the crystals during heating to ambient temperature.

Synthesis of [PPN][CN(HCN)$_3$]

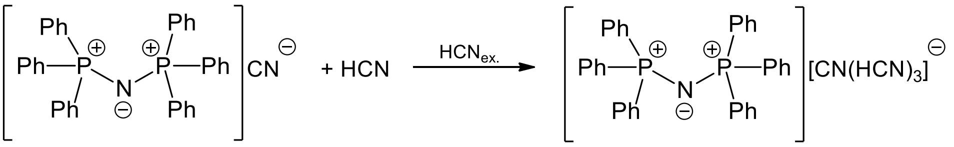

[PPN]CN (0.24 g, 0.43 mmol) was placed in a Schlenk tube and cooled to −12 °C. HCN (200 µL, 0.13 g, 4.93 mmol, 11 eq.), cooled to 0 °C, was added via a precooled syringe which led to a suspension. The mixture was slowly allowed to warm up to 0 °C until complete dissolution was observed. The liquid phase changed its color to yellowish/brownish. The mixture was cooled to −10 °C and crystals could be obtained after fifteen minutes. Crystals, suitable for X-Ray diffraction, could be separated via crystal picking and analysis of the single crystals revealed the formation of [PPN][CN(HCN)$_3$].

$C_{40}H_{33}N_5P_2$ (645.65 g/mol) **Raman** (laser: 633 nm, accumulation time: 20 s, 20 scans, 233 K, cm^{-1}) $\tilde{\nu}$ = 170 (1), 182 (1), 188 (1), 205 (1), 222 (1), 239 (2), 251 (1), 255 (1), 269 (1), 283 (1), 319 (1), 367 (1), 391 (1), 398 (1), 489 (1), 527 (1), 536 (1), 551 (1),

616 (2), 667 (3), 694 (1), 699 (1), 724 (1), 730 (1), 750 (1), 758 (1), 761 (1), 805 (1), 845 (1), 863 (1), 929 (1), 939 (1), 962 (1), 975 (1), 1002 (10), 1027 (3), 1057 (1), 1073 (1), 1077 (1), 1091 (1), 1113 (3), 1160 (1), 1165 (1), 1184 (1), 1192 (1), 1283 (1), 1314 (1), 1337 (1), 1384 (1), 1398 (1), 1436 (1), 1443 (1), 1484 (1), 1576 (1), 1589 (3), 1616 (1), 2055 (1), 2073 (2), 2560 (1), 2611 (1), 2960 (1), 2994 (1), 2998 (1), 3017 (1), 3043 (1), 3064 (2), 3081 (1), 3144 (1), 3150 (1), 3179 (1).

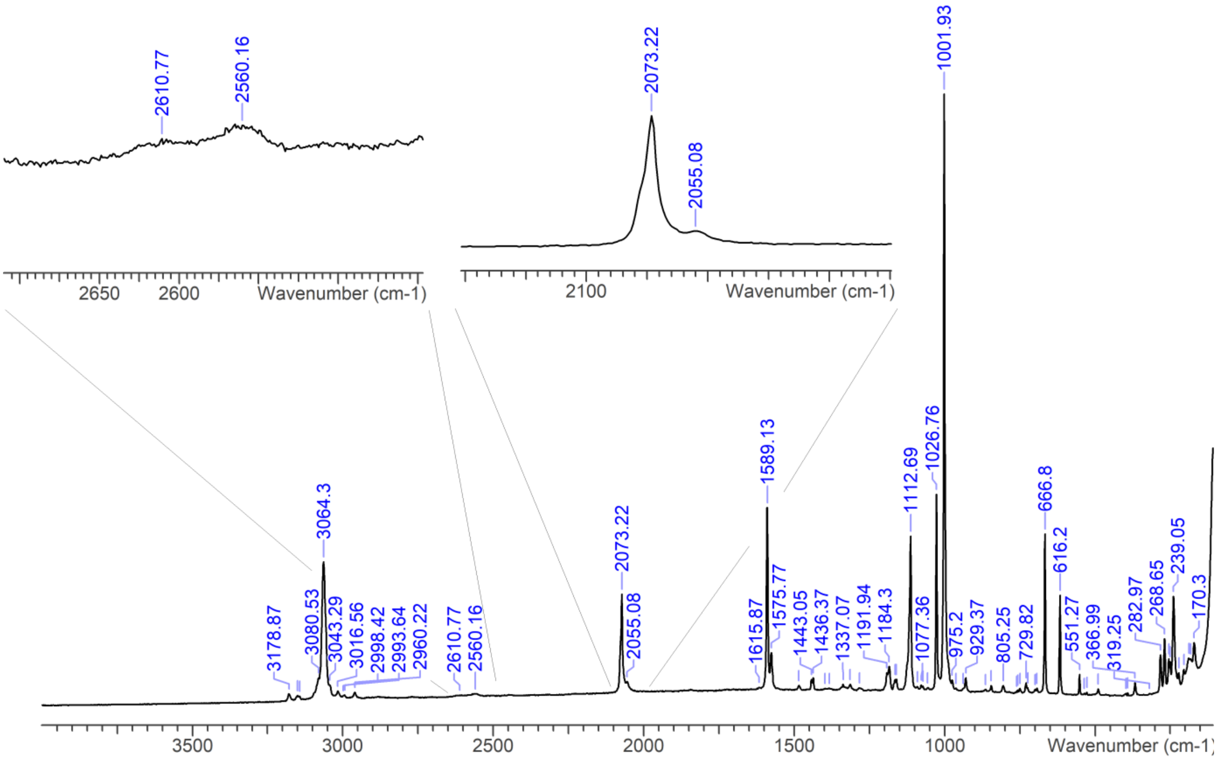

Figure S 5. Raman spectrum of [PPN][CN(HCN)$_3$].

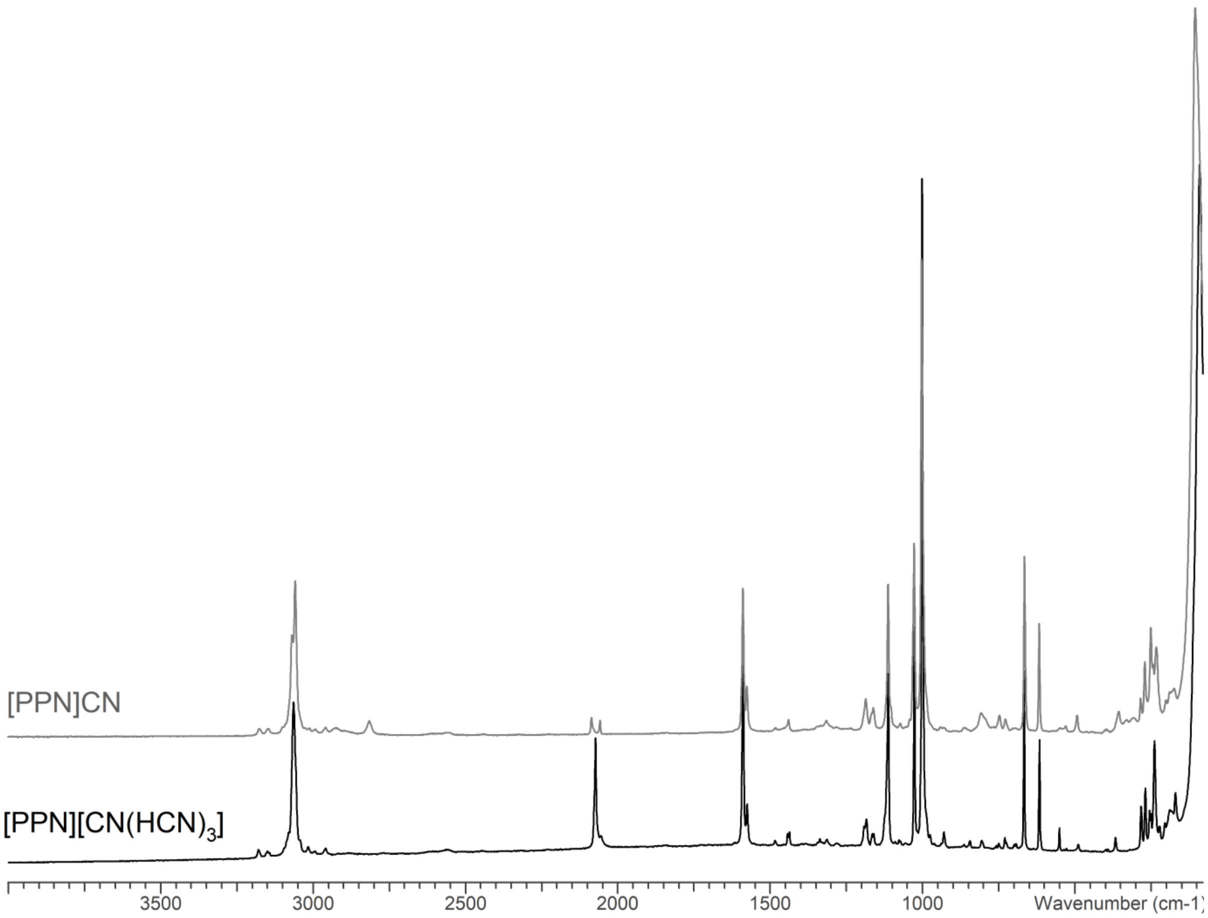

Figure S 6. Raman spectra of [PPN][CN(HCN)$_3$] (black) and reference material [PPN]CN (grey) for comparison.

[PPN][CN(DCN)$_X$]

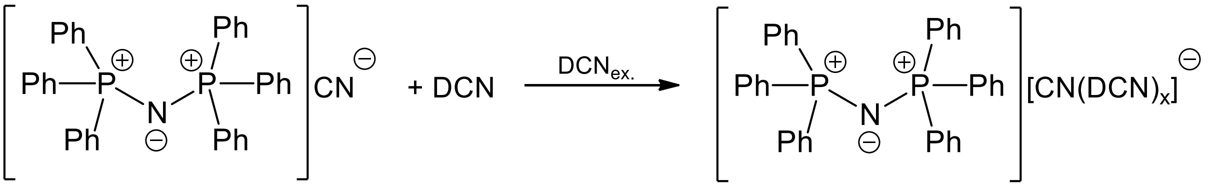

[PPN]CN (0.10 g, 0.17 mmol) was placed in a specially built Raman-tube and cooled to −20 °C. DCN (200 µL, excess), cooled to −12 °C, was added via a precooled syringe. The mixture was slowly warmed up until complete dissolution of the cyanide salt was observed. Color change of the liquid phase from colorless to yellowish could be observed when the salt started to dissolve. Once the entire solid had been dissolved, the tube was placed in a low-temperature stage for the Raman device[8], cooled to −15 °C. Crystal formation could be observed by slow and stepwise cooling to −19 °C. Unfortunately, the crystal could not be isolated of the Raman-tube for single-

crystal-X-Ray experiments. Further cooling of the mixture to −20 °C led to solidifying of the whole liquid. A Raman spectrum of the crystal at −19 °C was recorded.

Raman of the crystal (laser: 633 nm, accumulation time: 3 s, 20 scans, 254 K, cm^{-1}) $\tilde{\nu}$ = 173 (4), 240 (5), 251 (4), 306 (4), 365 (4), 424 (4), 494 (5), 557 (5), 617 (6), 667 (7), 710 (6), 730 (6), 801 (6), 864 (7), 952 (7), 1001 (10), 1028 (8), 1113 (8), 1235 (8), 1346 (8), 1444 (9), 1578 (9), 1590 (9), 1838 (9), 1906 (9), 2071 (8), 2202 (7), 2360 (7), 2505 (7), 2608 (6), 2785 (5), 3016 (4), 3061 (5), 3152 (4).

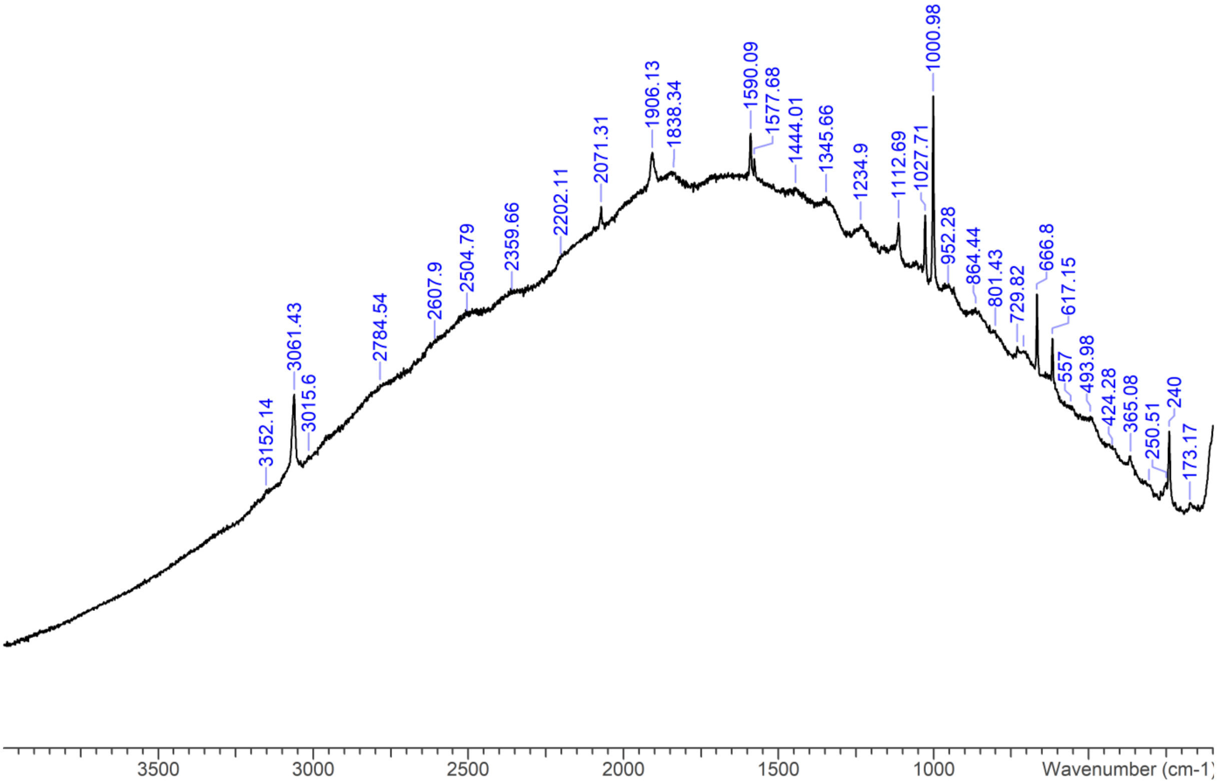

Figure S 7. Raman spectrum of the crystalline solid at −19 °C, obtained from the reaction mixture of [PPN]CN + DCN.

Synthesis of [PPh_4][$CN(HCN)_2$]

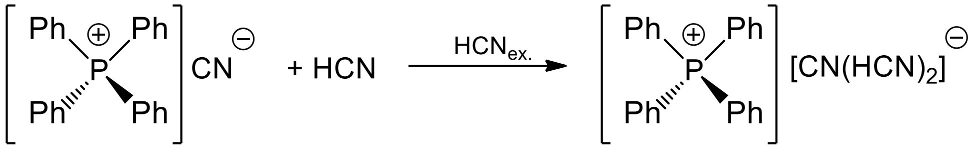

[PPh_4]CN (0.16 g, 0.44 mmol) was placed in a Schlenk tube and cooled to −20 °C. HCN (120 µL, 0.08 g, 3.06 mmol, 7 eq.), cooled to −10 °C, was added via a precooled syringe which led to a suspension. The mixture was slowly allowed to warm up until complete dissolution of the cyanide salt was observed. The liquid phase changed its color to yellowish/brownish. The mixture was slowly cooled to −20 °C and placed in a refrigerator. Crystal-formation could be observed within three hours, while the mixture became more viscous and changed the color to brownish. Single-crystals suitable for X-Ray diffraction could be separated from a brownish oily phase via crystal picking. Analysis revealed the formation of [PPh_4][$CN(HCN)_2$].

$C_{27}H_{22}N_3P$ (392.43 g/mol): **Raman** (laser: 633 nm, accumulation time: 10 s, 20 scans, 233 K, cm^{-1}) $\tilde{\nu}$ = 186 (1), 189 (1), 202 (2), 253 (2), 265 (1), 283 (1), 289 (1), 296 (1), 310 (1), 337 (1), 358 (1), 400 (1), 476 (1), 492 (1), 534 (1), 545 (1), 619 (1), 638 (1), 683 (2), 693 (1), 706 (1), 727 (1), 752 (1), 768 (1), 797 (1), 809 (1), 848 (1), 941 (1), 953 (1), 965 (1), 989 (1), 1005 (10), 1019 (1), 1029 (3), 1074 (1), 1102 (3), 1114 (1), 1154 (1), 1163 (1), 1187 (1), 1194 (1), 1242 (1), 1278 (1), 1293 (1), 1317 (1), 1342 (1), 1439 (1), 1487 (1), 1576 (1), 1590 (4), 2057 (1), 2081 (1), 2562 (1), 2960 (1), 2999 (1), 3014 (1), 3024 (1), 3068 (3), 3087 (1), 3150 (1).

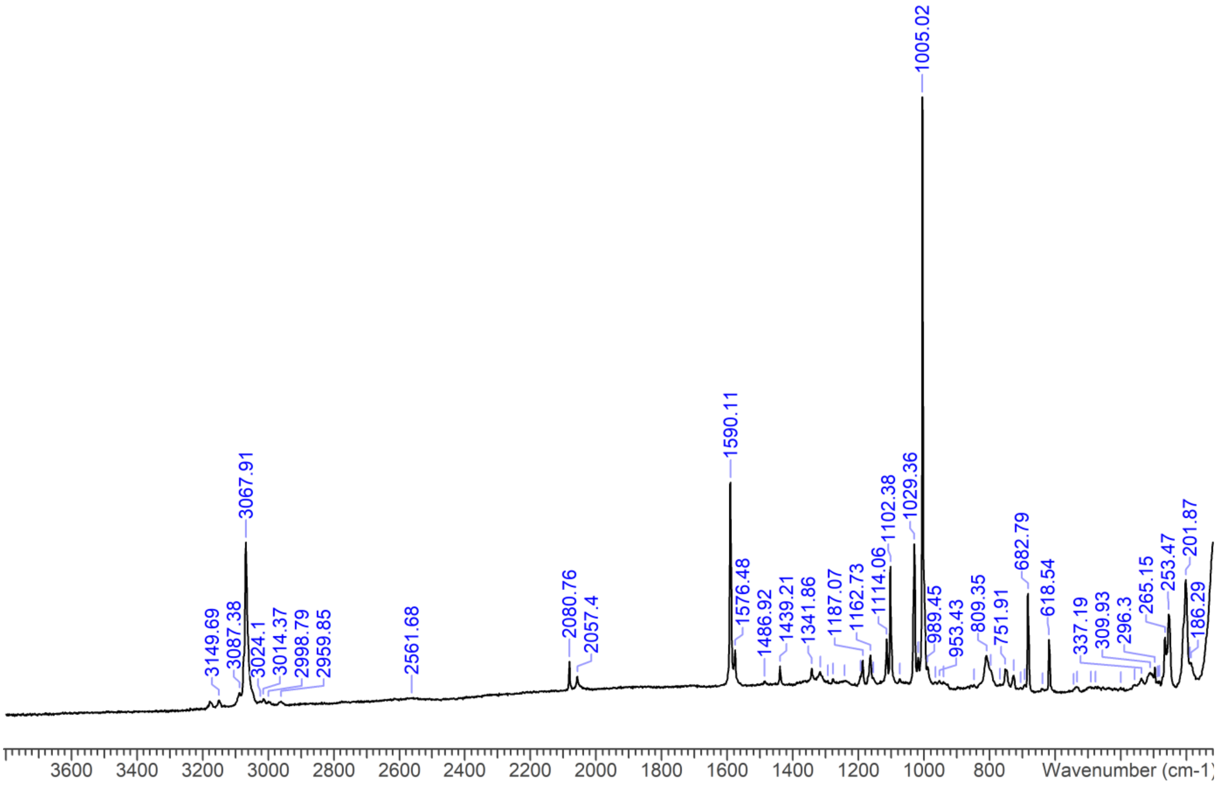

Figure S 8. Raman spectrum of $[PPh_4][CN(HCN)_2]$.

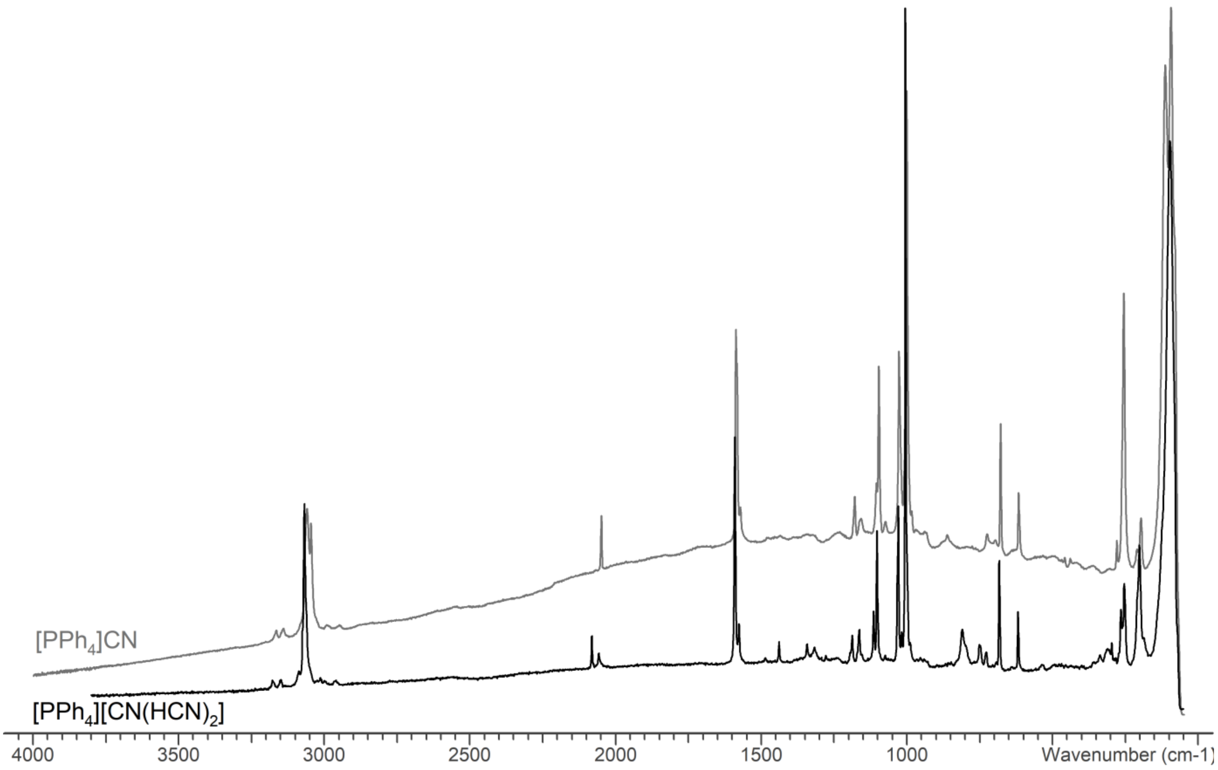

Figure S 9. Raman spectra of $[PPh_4][CN(HCN)_2]$ (black) and reference material $[PPh_4]CN$ (grey) for comparison.

[PPh_4][CN(DCN)$_x$]

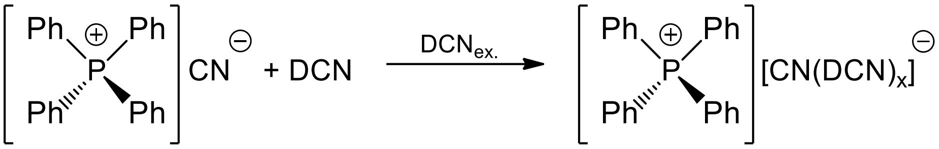

[PPh_4]CN (0.07 g, 0.19 mmol) was placed in a specially built Raman-tube and cooled to −20 °C. DCN (100 µL, excess), cooled to −12 °C, was added via a precooled syringe. The mixture was slowly warmed up until complete dissolution of the cyanide salt was observed. Color change of the liquid phase from colorless to yellowish could be observed when the salt started to dissolve. Once the entire solid had been dissolved, the tube was placed in a low-temperature stage for the Raman device[8], cooled to −18 °C. Unfortunately, slow and stepwise cooling to −29 °C did not produce any crystals. At −30 °C the whole liquid solidified but a Raman spectrum of the solid could not be recorded (strong fluorescence), so Raman spectra of the liquid reaction mixture at −28 °C were recorded.

Raman of liquid phase (laser: 633 nm, accumulation time: 6 s, 15 scans, 245 K, cm^{-1}) $\tilde{\nu}$ = 201 (3), 252 (4), 292 (3), 313 (3), 365 (4), 423 (4), 507 (4), 561 (5), 618 (5), 637 (5), 681 (6), 727 (6), 800 (6), 867 (6), 962 (7), 1003 (9), 1030 (8), 1057 (7), 1101 (8), 1114 (7), 1150 (8), 1167 (7), 1243 (8), 1348 (8), 1461 (9), 1579 (9), 1591 (10), 1718 (9), 1906 (10), 1986 (10), 2070 (10), 2266 (9), 2385 (8), 2519 (8), 2639 (8), 2833 (7), 2964 (7), 3072 (7), 3173 (6).

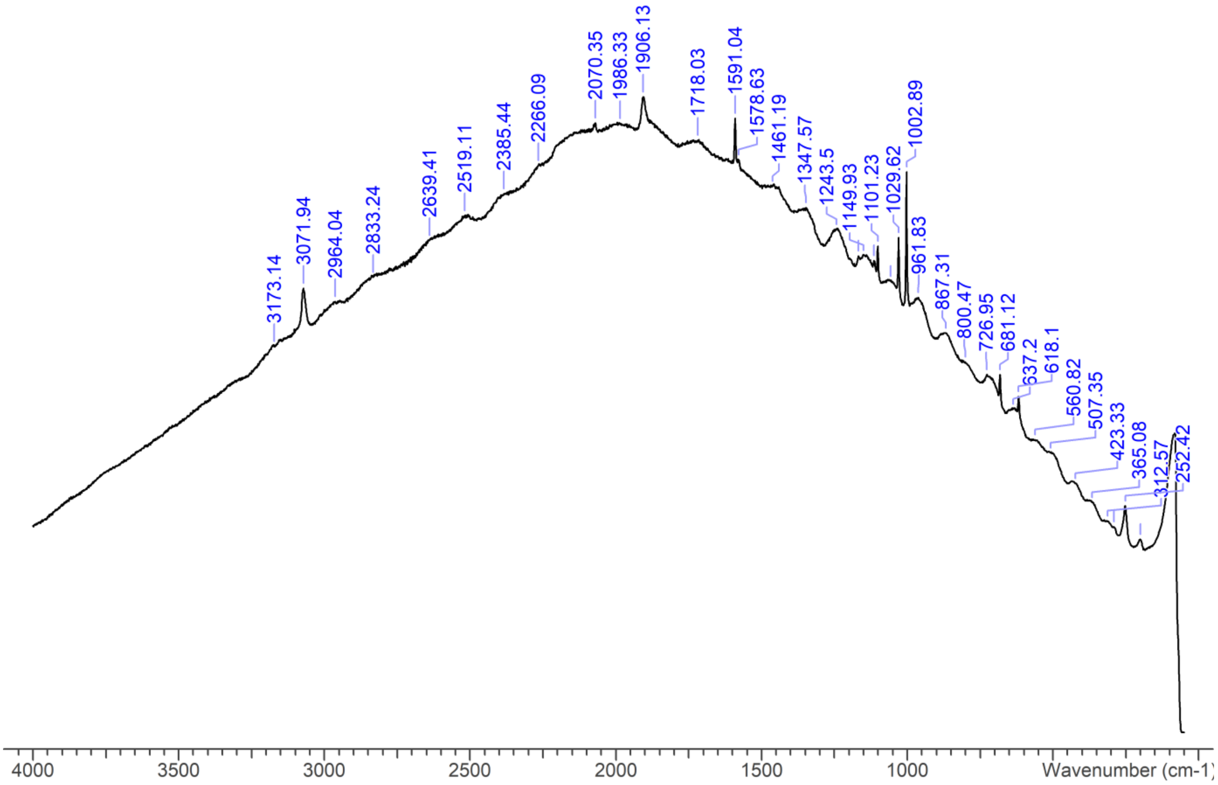

Figure S 10. Raman spectrum of the liquid phase of the reaction of $[PPh_4]CN$ and DCN at −28 °C.

NMR spectroscopic studies for $[PPh_4]CN$ + HCN

In an additional nmr experiment, we tried to study the formation of $[CN(HCN)_x]^-$ species (x = 1, 2, 3…). A nmr-tube was charged with $[PPh_4]CN$ (0.06 g, 0.16 mmol) and an excess amount of HCN (400 µL, 10.20 mmol). The suspension (−10 °C) was allowed to warm up until dissolution of the cyanide salt was achieved. The yellowish liquid was cooled to −12 °C (further cooling led to solidifying of the mixture) and nmr spectroscopic investigations were performed. Unfortunately, we could only observe HCN and the solvated CN^- as shown in the nmr spectra below.

^{1}H NMR (261 K, no deuterated solvent, 250.13 MHz) δ = 8.26 – 7.91 (m, 20 H, $P(C_6H_5)_4$); 4.73 – 4.63 (m, *H*CN). **^{13}C{^{1}H} NMR** (261 K, no deuterated solvent, 62.9 MHz) δ = 169.2 (s, *C*N$^-$); 135.8 (d, *p-C*, $^4J(^{13}C–^{31}P)$ = 3 Hz); 134.9 (d, *m-C*, $^3J(^{13}C–^{31}P)$ = 11 Hz); 130.7 (d, *o-C*, $^2J(^{13}C–^{31}P)$ = 13 Hz); 117.9 (d, *i-C*, $^1J(^{13}C–^{31}P)$ = 89 Hz); 111.8 (br, H*C*N). **^{14}N{^{1}H} NMR** (261 K, no deuterated solvent, 18.1 MHz) δ = −127.9 (br, HC*N*).

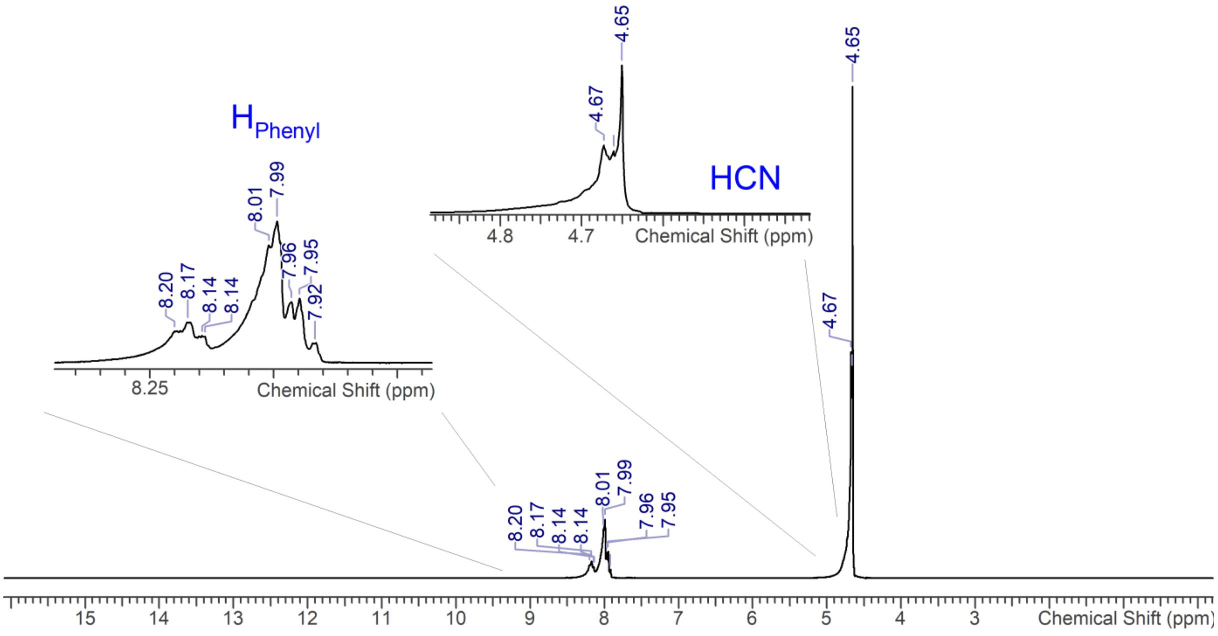

Figure S 11. ^{1}H NMR Spectrum of the mixture $[Ph_4P]CN$ + HCN. The spectrum was calibrated externally.

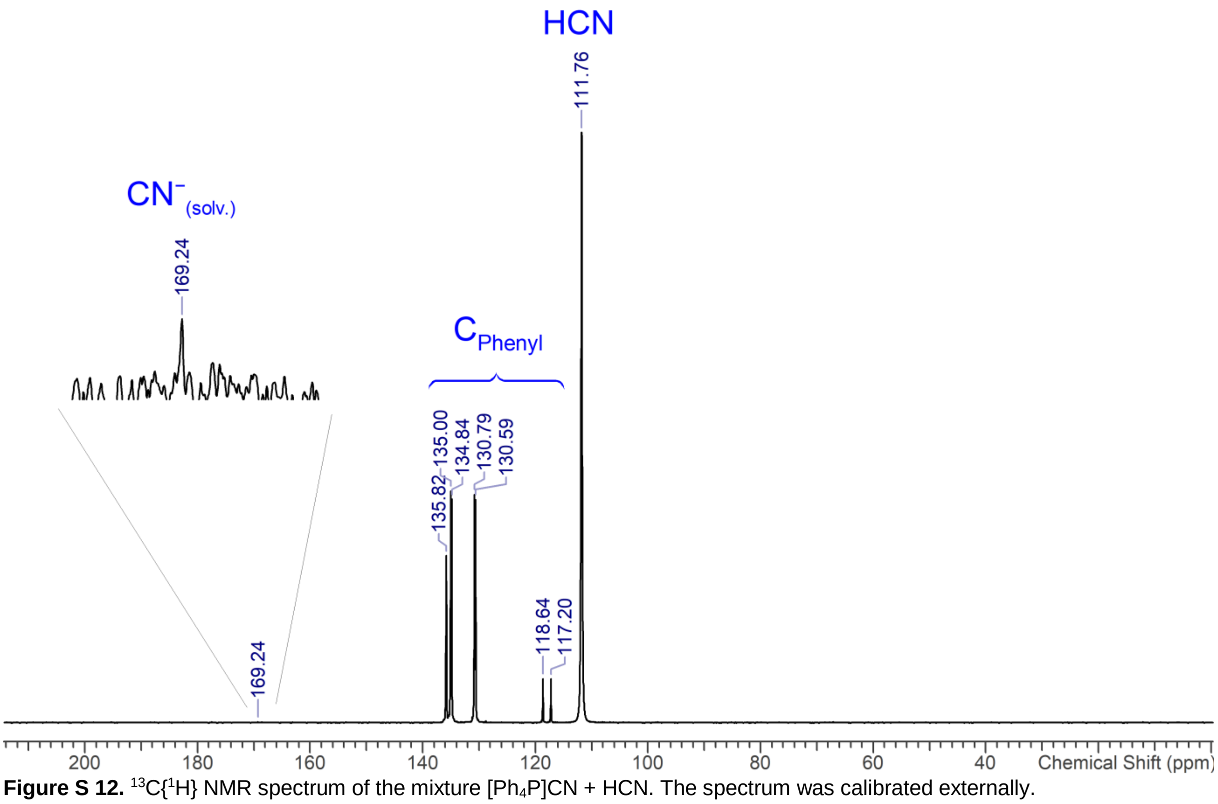

Figure S 12. $^{13}C\{^{1}H\}$ NMR spectrum of the mixture $[Ph_4P]CN$ + HCN. The spectrum was calibrated externally.

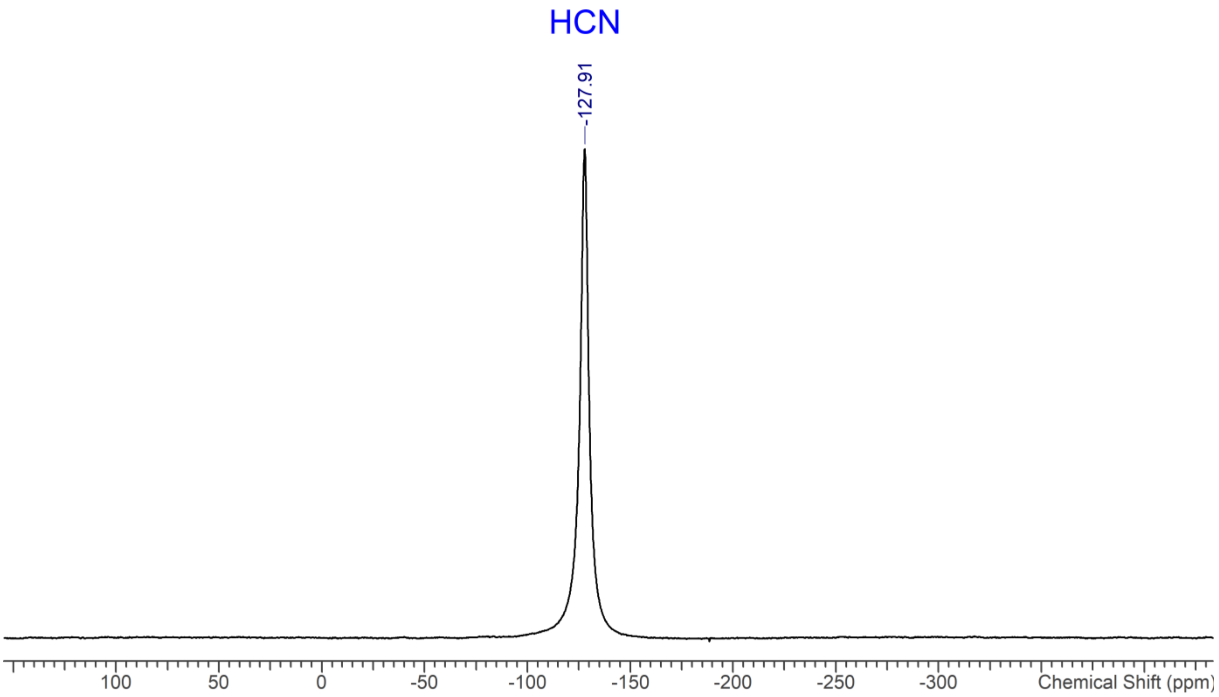

Figure S 13. $^{14}N\{^{1}H\}$ NMR spectrum of the mixture $[Ph_4P]CN$ + HCN. The spectrum was calibrated externally.

Attempted, not successful syntheses

[WCC][CN(HCN)$_x$] using TMS-CN

$$[^{n}Pr_3NMe]CN + TMS\text{-}CN + MeOH \xrightarrow[\quad /\!/ \quad]{TMS\text{-}CN_{ex.}} [^{n}Pr_3NMe][CN(HCN)_x] + TMS\text{-}OMe$$

[^{n}Pr$_3$NMe]CN (0.22 g, 1.20 mmol) was suspended in TMS–CN (4 mL, 3.18 g, 31.96 mmol, 26 eq.). MeOH (22 droplets) was added dropwise until dissolution of the cyanide salt was observed. The light yellowish liquid was filtered with a glass frit and placed in the refrigerator at 5 °C. According to X-Ray analysis, crystals of the starting material [^{n}Pr$_3$NMe]CN could be obtained from the reaction mixture after five days.

$$[PPh_4]CN + TMS\text{-}CN + MeOH \xrightarrow[\quad /\!/ \quad]{TMS\text{-}CN_{ex.}} [PPh_4][CN(HCN)_x] + TMS\text{-}OMe$$

[PPh$_4$]CN (0.20 g, 0.54 mmol) was suspended in TMS–CN (2 mL, 1.59 g, 15.98 mmol, 30 eq.). MeOH (1 mL) was added dropwise until dissolution of the cyanide salt was observed. The light yellowish liquid was filtered with a glass frit and placed in the refrigerator at 5 °C. A black precipitate (presumably a polymeric product) was observed in the mixture after five days which was not further analyzed.

[WCC][CN(HCN)$_x$] using unsymmetrical cations

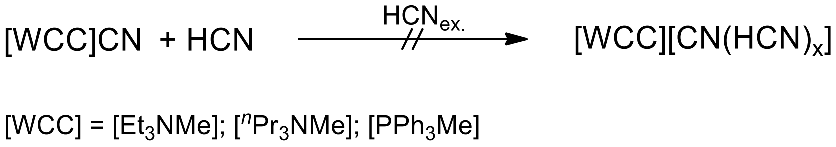

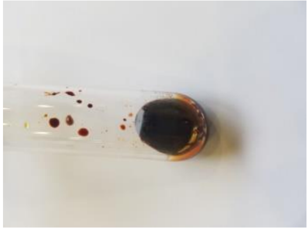

[WCC]CN (0.4 mmol) ([WCC] = [Et$_3$NMe]; [^{n}Pr$_3$NMe]; [Ph$_3$PMe]) was placed in a Schlenk tube and cooled to −10 °C. HCN (120 µL, 0.08 g, 3.06 mmol, 7 eq.), cooled to −12 °C, was added via a precooled syringe. The mixture was slowly allowed to warm up until complete dissolution of the cyanide salt was achieved. Colour change of the liquid phase from colorless to yellowish/brownish could be observed when the cyanide dissolved. Once the entire solid was dissolved, the mixture was slowly cooled to −20 °C and the reaction vessel was placed in a refrigerator overnight. Unfortunately, no crystals formed in the brown oil.

Annotation: No further analytics of the samples were recorded. Raman spectra could not be recorded due to strong fluorescence of the samples.

4. Computational Details

All computations were carried out using Gaussian09[9] as well as the standalone version of NBO 6.0.[10–13]

Methods. We started the project with an evaluation of the applied computational method for the $[CN(HCN)_n]^-$ (n = 1 - 3) ion. For this reason, we used the following DFT methods: PBE0 including Grimme's dispersion model D3BJ and M06 as well as the following ab initio methods: MP2 and CCSD(T) in conjunction with the aug-cc-pVDZ and aug-cc-pVTZ basis sets. As can be seen from table S1 - S5, for all methods a good qualitative as well as quantitative agreement could be found, so that only PBE0 and CCSD(T) were applied for the $[CN(HCN)_2]^-$ ion and for $[CN(HCN)_3]^-$ only PBE0 with the aug-cc-pVTZ base set. We would like to point out that with regard to the monomer also agreement with experimental as well as theoretical data was found.[14–19] In addition, to get better structural data, we used the PCM and SMD method utilizing different solvents to account for charge compensation. As expected, the structural data using PCM or SMP are closer to the Xray data, while the computed gas phase energies agree much better to the experimentally observed gas phase data.

Structure. All structures were fully optimized and confirmed as minima by frequency analyses (NIMAG = 0). Cartesian coordinates of all considered species can be obtained from the authors.

Population analysis. Partial charges, Lewis representations, delocalization and polarization effects etc. were computed using the NBO **Figure S16 and S17**. Potential of rotation (6.0 program (NBO, NRT, NBO deletion) at the PBE0/aug-cc-pVTZ level of theory.

Note. We would like to point out that all solvates of the type $[CN(HCN)_n]^-$ have very flat potentials for the bridging H–C and H–N distances as well as angles and dihedral angles. Therefore, the structure at ambient temperatures can be considered to be highly dynamic.

Although the calculated gas phase energy data agree very well with the experimental data, it is difficult to compare the structural data with one another, even if the thermodynamically favored gas phase structural motifs are also observed in the solid state (best isomer). However, there are significant differences in terms of linearity (dimer, linear in the gas phase versus non-linear in the solid state) and planarity (trimer, planar in the gas phase versus non-planar in the solid state) due to very soft potentials for bending/distortion. Hence weak cation / anion or anion / anion interactions, which are not taken into account in the gas phase calculations neither in the PCM/SMD computations, play an essential role.

Calibration - Comparison of computed and experimentally observed enthalpies

$CN^- + n\ HCN \rightarrow [CN(HCN)_n]^-$

$\Delta_r H°$(298 K) in kcal/mol

**reference**	n = 1	2	3
A	-20.7 ± 1.0	-16.4 ± 1.0	-12.6 ± 1.0
B	-20.6 ±1.6		
C	-21.7±3.5		
**our computation** (level used for all species)			
pbe-aug-cc-pvtz	-23.02 NC-H-CN<br>-21.35 CN-H-NC<br>-20.25 CN-H-NC<br>-23.59 NC-H-NC	-18.69<br>best isomer	-12.96<br>best isomer

(A) M. Meot-Ner, S. M. Cybulski, S. Scheiner, J. F. Liebman, *J. Phys. Chem.* **1988**, *92*, 2738–2745.
(B) S. A. Chacko, I. H. Krouse, L. A. Hammad, P. G. Wenthold, *J. Am. Soc. Mass Spectrom.* **2006**, *17*, 51–55.
(C) J. W. Larson, T. B. McMahon, *J. Am. Chem. Soc.* **1987**, *109*, 6230–6236.

For the reaction

2 HCN → 2 HCCH + N2

	$\Delta_f H°$(298 K)
ATCT data:	–7.24 kJ/mol (see https://atct.anl.gov/)
our computed Pbe/aug-cc-pvTZ:	–7.49 kJ/mol

Monosolvate $[CN(HCN)_1]^-$

dz = aug_cc_pVDZ

tz = aug_cc_pVTZ

Table S 1. Absolute energies in a.u.

HCN	pbe_dz	pbe_tz	m06_dz	m06_tz	mp2_dz	mp2_tz	ccsd(t)_dz	ccsd(t)_tz
**E0**	-93.321897	-93.344219	-93.401819	-93.424003	-93.183659	-93.259750	-93.204711	-93.281167
**H298**	-93.302074	-93.324305	-93.381838	-93.403920	-93.164528	-93.240457	-93.185504	-93.261759
**G298**	-93.324950	-93.347142	-93.404694	-93.426738	-93.187487	-93.263371	-93.208463	-93.284666
**Nimag**	**0**	**0**	**0**	**0**	**0**	**0**	**0**	**0**
**PG**	Coov	Coov	Coov	Coov	Coov	Coov	Coov	Coov
**CN^-**	**pbe_dz**	**pbe_tz**	**m06_dz**	**m06_tz**	**mp2_dz**	**mp2_tz**	**ccsd(t)_dz**	**ccsd(t)_tz**
**E0**	-92.755709	-92.774938	-92.839897	-92.860898	-92.620710	-92.693083	-92.640527	-92.713105
**H298**	-92.747535	-92.766741	-92.831647	-92.852623	-92.612974	-92.685275	-92.632627	-92.705134
**G298**	-92.769890	-92.789078	-92.853996	-92.874955	-92.635370	-92.707644	-92.655013	-92.727494
**Nimag**	**0**	**0**	**0**	**0**	**0**	**0**	**0**	**0**
**PG**	Coov	Coov	Coov	Coov	Coov	Coov	Coov	Coov
**$[NC-H-CN]^-$**	**pbe_dz**	**pbe_tz**	**m06_dz**	**m06_tz**	**mp2_dz**	**mp2_tz**	**ccsd(t)_dz**	**ccsd(t)_tz**
**E0**	-186.113771	-186.153197	-186.273976	-186.314754	-185.834349	-185.981589	185.8735143	-186.021659
**H298**	-186.089139	-186.128448	-186.249142	-186.289815	-185.810461	-185.957676	-185.849233	-185.997354
**G298**	-186.120411	-186.159635	-186.280112	-186.320699	-185.841637	-185.988905	-185.880247	-186.028416
**Nimag**	**1**	**1**	**1**	**1**	**1**	**1**	**1**	**1**
**PG**	Dooh	Dooh	Dooh	Dooh	Dooh	Dooh	Dooh	Dooh
**$[NC-H-CN]^-$**	**pbe_dz**	**pbe_tz**	**m06_dz**	**m06_tz**	**mp2_dz**	**mp2_tz**	**ccsd(t)_dz**	**ccsd(t)_tz**
**E0**	-186.115945	-186.156333	-186.277317	-186.319413	-185.839076	-185.987326	-<br>185.8790731	-186.028194
**H298**	-186.087677	-186.127738	-186.248556	-186.290395	-185.811001	-185.959124	-185.850579	-185.999581
**G298**	-186.120466	-186.160550	-186.281293	-186.323185	-185.844076	-185.992271	-185.883602	-186.032593

**Nimag**	**0**	**0**	**0**	**0**	**0**	**0**	**0**	**0**
**PG**	Coov	Coov	Coov	Coov	Coov	Coov	Coov	Coov
**[CN-H-NC]$^-$**	**pbe_dz**	**pbe_tz**	**m06_dz**	**m06_tz**	**mp2_dz**	**mp2_tz**	**ccsd(t)_dz**	**ccsd(t)_tz**
**E0**	-186.110008	-186.149697	-186.274662	-186.314714	-185.828130	-185.975992	- 185.8707663	-186.019056
**H298**	-186.084366	-186.125064	-186.248331	-186.288749	-185.804267	-185.952006	-185.846644	-185.994795
**G298**	-186.116519	-186.156171	-186.279280	-186.320140	-185.835375	-185.983164	-185.877685	-186.025819
**Nimag**	**0**	**1**	**0**	**0**	**1**	**1**	**1**	**1**
**PG**	Dooh	Dooh	Dooh	Dooh	Dooh	Dooh	Dooh	Dooh
**[CN-H-NC]$^-$**	**pbe_dz**	**pbe_tz**	**m06_dz**	**m06_tz**	**mp2_dz**	**mp2_tz**	**ccsd(t)_dz**	**ccsd(t)_tz**
**E0**	-186.110008	-186.149735	-186.274662	-186.314713	-185.828263	-185.976247	- 185.8713293	-186.019809
**H298**	-186.084352	-186.123316	-186.248325	-186.288744	-185.802108	-185.949644	-185.844109	-185.992268
**G298**	-186.117126	-186.155657	-186.279938	-186.320795	-185.834393	-185.981932	-185.876402	-186.024479
**Nimag**	**0**	**0**	**0**	**0**	**0**	**0**		**0**
**PG**	Coov	Coov	Coov	Coov	Coov	Coov	Coov	Coov
**[NC-H-NC]$^-$**	**pbe_dz**	**pbe_tz**	**m06_dz**	**m06_tz**	**mp2_dz**	**mp2_tz**	**ccsd(t)_dz**	**ccsd(t)_tz**
**E0**	-186.117148	-186.157484	-186.279283	-186.321319	-185.840176	-185.988383	-185.881048	-186.029975
**H298**	-186.088495	-186.128647	-186.250402	-186.292215	-185.811906	-185.959971	-185.852468	-186.001240
**G298**	-186.121300	-186.161579	-186.283093	-186.325039	-185.845112	-185.993127	-185.885577	-186.034287
**Nimag**	**0**	**0**	**0**	**0**	**0**	**0**	**0**	**0**
**PG**	Coov	Coov	Coov	Coov	Coov	Coov	Coov	Coov

Table S 2. Relative energies in kcal/mol.

ΔE0K

rel. Isomers	pbe_dz	pbe_tz	m06_dz	m06_tz	mp2_dz	mp2_tz	ccsd(t)_dz	ccsd(t)_tz
$[NC\text{-}HCN]^-$	0.00	0.00	0.00	0.00	0.00	0.00	0.00	0.00
$[CN\text{-}HNC]^-$	3.73	4.14	1.67	2.95	6.79	6.95	4.86	5.26
$[NC\text{-}HNC]^-$	-0.75	-0.72	-1.23	-1.20	-0.69	-0.66	-1.24	-1.12

ΔH298K

rel. Isomers	pbe_dz	pbe_tz	m06_dz	m06_tz	mp2_dz	mp2_tz	ccsd(t)_dz	ccsd(t)_tz
$[NC\text{-}HCN]^-$	0.00	0.00	0.00	0.00	0.00	0.00	0.00	0.00
$[CN\text{-}HNC]^-$	2.09	2.77	0.14	1.04	5.58	5.95	4.06	4.59
$[NC\text{-}HNC]^-$	-0.51	-0.57	-1.16	-1.14	-0.57	-0.53	-1.19	-1.04

ΔG298K

rel. Isomers	pbe_dz	pbe_tz	m06_dz	m06_tz	mp2_dz	mp2_tz	ccsd(t)_dz	ccsd(t)_tz
$[NC\text{-}HCN]^-$	0.00	0.00	0.00	0.00	0.00	0.00	0.00	0.00
$[CN\text{-}HNC]^-$	2.10	3.07	0.85	1.50	6.08	6.49	4.52	5.09
$[NC\text{-}HNC]^-$	-0.52	-0.65	-1.13	-1.16	-0.65	-0.54	-1.24	-1.06

Table S 3. Reaction energies in kcal/mol.

HCN + CN⁻ → [NC-H-CN]⁻_Dooh	pbe_dz	pbe_tz	m06_dz	m06_tz	mp2_dz	mp2_tz	ccsd(t)_dz	ccsd(t)_tz
**ΔE0**	-22.69	-21.36	-20.24	-18.73	-18.81	-18.04	-17.74	-17.19
**ΔH298**	-24.81	-23.47	-22.38	-20.88	-20.68	-20.05	-19.52	-19.11
**ΔG298**	-16.05	-14.69	-13.44	-11.93	-11.78	-11.23	-10.52	-10.20
HCN + CN⁻ → [NC-H-CN]⁻_Cooh	pbe_dz	pbe_tz	m06_dz	m06_tz	mp2_dz	mp2_tz	ccsd(t)_dz	ccsd(t)_tz
**ΔE0**	-24.06	-23.33	-22.34	-21.66	-21.78	-21.64	-21.23	-21.29
**ΔH298**	-23.89	-23.02	-22.01	-21.24	-21.02	-20.95	-20.36	-20.51
**ΔG298**	-16.08	-15.27	-14.18	-13.49	-13.32	-13.34	-12.63	-12.82
HCN + CN⁻ → [CN-H-NC]⁻_Dooh	pbe_dz	pbe_tz	m06_dz	m06_tz	mp2_dz	mp2_tz	ccsd(t)_dz	ccsd(t)_tz
**ΔE0**	-20.33	-19.16	-20.67	-18.71	-14.91	-14.53	-16.02	-15.55
**ΔH298**	-21.81	-21.35	-21.87	-20.21	-16.80	-16.49	-17.89	-17.51
**ΔG298**	-13.60	-12.52	-12.92	-11.58	-7.86	-7.62	-8.92	-8.57
HCN + CN⁻ → [CN-H-NC]⁻_Cooh	pbe_dz	pbe_tz	m06_dz	m06_tz	mp2_dz	mp2_tz	ccsd(t)_dz	ccsd(t)_tz
**ΔE0**	-20.33	-19.19	-20.67	-18.71	-14.99	-14.69	-16.37	-16.02
**ΔH298**	-21.80	-20.25	-21.86	-20.21	-15.44	-15.00	-16.30	-15.92
**ΔG298**	-13.98	-12.20	-13.33	-11.99	-7.24	-6.85	-8.11	-7.73
HCN + CN⁻ → [CN-H-NC]⁻_Cooh	pbe_dz	pbe_tz	m06_dz	m06_tz	mp2_dz	mp2_tz	ccsd(t)_dz	ccsd(t)_tz
**ΔE0**	-24.81	-24.05	-23.57	-22.85	-22.47	-22.31	-22.47	-22.40
**ΔH298**	-24.40	-23.59	-23.17	-22.38	-21.59	-21.49	-21.55	-21.55
**ΔG298**	-16.60	-15.91	-15.31	-14.65	-13.97	-13.88	-13.87	-13.88

Table S 4. Structural data (distances in Å).

HCN	pbe_dz	pbe_tz	m06_dz	m06_tz	mp2_dz	mp2_tz	ccsd(t)_dz	ccsd(t)_tz
H-C	1.075	1.068	1.074	1.066	1.078	1.065	1.082	1.067
C-N	1.156	1.146	1.152	1.142	1.183	1.167	1.176	1.160
**CN^-**	**pbe_dz**	**pbe_tz**	**m06_dz**	**m06_tz**	**mp2_dz**	**mp2_tz**	**ccsd(t)_dz**	**ccsd(t)_tz**
C-N	1.182	1.171	1.178	1.168	1.208	1.191	1.202	1.185
**$[NC-H-CN]^-$_Dooh**	**pbe_dz**	**pbe_tz**	**m06_dz**	**m06_tz**	**mp2_dz**	**mp2_tz**	**ccsd(t)_dz**	**ccsd(t)_tz**
H-C	1.398	1.397	1.401	1.400	1.397	1.392	1.402	1.395
**C-C**	**2.795**	**2.794**	**2.801**	**2.800**	**2.794**	**2.783**	**2.805**	**2.791**
C-N	1.170	1.160	1.166	1.156	1.197	1.181	1.190	1.174
**$[NC-H-CN]^-$_Coov**	**pbe_dz**	**pbe_tz**	**m06_dz**	**m06_tz**	**mp2_dz**	**mp2_tz**	**ccsd(t)_dz**	**ccsd(t)_tz**
H-C	1.168	1.149	1.152	1.134	1.139	1.122	1.137	1.121
H-C	1.781	1.828	1.839	1.884	1.906	1.923	1.937	1.946
**C-C**	**2.949**	**2.977**	**2.992**	**3.019**	**3.045**	**3.045**	**3.075**	**3.066**
C-N	1.162	1.152	1.158	1.148	1.187	1.171	1.180	1.164
C-N	1.176	1.166	1.173	1.163	1.906	1.188	1.197	1.181
**$[CN-H-NC]^-$_Dooh**	**pbe_dz**	**pbe_tz**	**m06_dz**	**m06_tz**	**mp2_dz**	**mp2_tz**	**ccsd(t)_dz**	**ccsd(t)_tz**
H-N	1.268	1.268	1.272	1.272	1.272	1.267	1.275	1.269
**N-N**	**2.536**	**2.535**	**2.544**	**2.544**	**2.544**	**2.534**	**2.549**	**2.538**
C-N	1.175	1.165	1.171	1.162	1.198	1.183	1.193	1.178
**$[CN-H-NC]^-$_Coov**	**pbe_dz**	**pbe_tz**	**m06_dz**	**m06_tz**	**mp2_dz**	**mp2_tz**	**ccsd(t)_dz**	**ccsd(t)_tz**
H-N	1.262	1.183	1.267	1.267	1.161	1.140	1.126	1.112
H-N	1.273	1.370	1.277	1.277	1.416	1.439	1.492	1.502
**N-N**	**2.536**	**2.553**	**2.543**	**2.544**	**2.577**	**2.579**	**2.618**	**2.614**
C-N	1.175	1.163	1.171	1.162	1.195	1.179	1.190	1.175
C-N	1.175	1.166	1.172	1.162	1.200	1.186	1.196	1.181
**$[CN-H-CN]^-$_Coov**	**pbe_dz**	**pbe_tz**	**m06_dz**	**m06_tz**	**mp2_dz**	**mp2_tz**	**ccsd(t)_dz**	**ccsd(t)_tz**
H-C	1.154	1.139	1.149	1.129	1.132	1.117	1.133	1.117
H-N	1.657	1.693	1.682	1.730	1.768	1.779	1.180	1.788
**N-C**	**2.812**	**2.833**	**2.830**	**2.859**	**2.900**	**2.895**	**2.914**	**2.905**
C-N	1.162	1.151	1.158	1.147	1.186	1.170	1.180	1.164

C-N	1.179	1.169	1.682	1.166	1.204	1.189	1.199	1.183

Table S 5. H-bond analysis: Connectivity vs. relative energy in kcal/mol.

**pbepbo_tz**<br>monomer<br>pbe_tz<br>isomer	**symmetry**	**connectivity**		**number of HN**	**bonds CH**	**number of HN bridge**	**bonds HC bridge**	**ΔE0**	**ΔH298**	**ΔG298**
**[NC-H-CN]$^-$**	Coov	NC-H	CN	0	1	0	1	**0.72**	**0.57**	**0.65**
**[CN-H-NC]$^-$**	Coov	CN-H	NC	1	0	1	0	**4.86**	**3.35**	**3.72**
**[NC-H-NC]$^-$**	Coov	NC-H	NC	0	1	1	0	**0.00**	**0.00**	**0.00**
**ccsd(t)_tz**<br>Monomer<br>pbe_tz<br>Isomer	**symmetry**	**connectivity**		**number of HN**	**bonds CH**	**number of HN_bridge**	**bonds HC_bridge**	**ΔE0**	**ΔH298**	**ΔG298**
**[NC-H-CN]$^-$**	Coov	NC-H	CN	0	1	0	1	**1.12**	**1.04**	**1.06**
**[CN-H-NC]$^-$**	Coov	CN-H	NC	1	0	1	0	**6.38**	**5.63**	**6.15**
**[NC-H-NC]$^-$**	Coov	NC-H	NC	0	1	1	0	**0.00**	**0.00**	**0.00**

Disolvate $[CN(HCN)_2]^-$

Table S 6. Absolute energies in a.u, relative energies in kcal/mol at pbe0/aug-cc-pVTZ.

isomer	symmetry	connectivity			number of HN	bonds CH	number of HN_bridge	bonds HC_bridge	E0	H298	G298	ΔE0	ΔH298	ΔG298
y1	C2v	NCH<br>NC<br>NCH			0	2	2	0	-279.527852	-279.477945	-279.524076	3.20	3.01	1.43
y2	C2v	NCH<br>CN<br>NCH			0	2	0	2	-279.524537	-279.474641	-279.520421	5.28	5.08	3.72
y3	C2v	CNH<br>CN<br>CNH			2	0	0	2	-279.497702	-279.448463	-279.492760	22.12	21.51	21.08
y4	C2v	CNH<br>NC<br>CNH			2	0	2	0	-279.501601	-279.452359	-279.497151	19.68	19.06	18.32
t1	Cs	NCH<br>NC<br>CNH			1	1	2	0	-279.516655	-279.467300	-279.512526	10.23	9.68	8.67
lin1	Coov	NCH	**CN**	HCN	0	2	1	1	**-279.532957**	**-279.482734**	**-279.526347**	**0.00**	0.00	0.00
lin2	Coov	NCH	**CN**	HNC	1	1	1	1	-279.522838	-279.473798	-279.509426	6.35	5.61	10.62
lin3	Coov	NCH	**NC**	HNC	1	1	1	1	-279.523482	-279.474682	-279.517637	5.95	5.05	5.47
lin4	Coov	CNH	NCH	**NC**	1	1	2	0	-279.510176	-279.462366	-279.505135	14.30	12.78	13.31
lin5	Coov	NCH	NCH	**NC**	0	2	2	0	-279.523708	-279.474380	-279.517957	5.80	5.24	5.26

Table S 7. Absolute energies in a.u, relative energies in kcal/mol at CCSD(T)/aug-cc-pVTZ.

isomer	symmetry	connectivity	number of HN	bonds CH	number of HN_bridge	bonds HC_bridge	E0	ΔE0
y1	C2v	NCH<br>NC<br>NCH	0	2	2	0	-279.336594	2.53
y2	C2v	NCH<br>CN<br>NCH	0	2	0	2	-279.332484	5.11
y3	C2v	CNH<br>CN<br>CNH	2	0	0	2	-279.306888	21.17
y4	C2v	CNH<br>NC<br>CNH	2	0	2	0	-279.306887	21.17
t1	Cs	NCH<br>NC<br>CNH	1	1	2	0	-279.32316	10.96
lin1	Coov	NCH **CN** HCN	0	2	1	1	**-279.340625**	**0.00**
lin2	Coov	NCH **CN** HNC	1	1	1	1	-279.328642	7.52
lin3	Coov	NCH **NC** HNC	1	1	1	1	-279.329028	7.28
lin4	Coov	CNH NCH **NC**	1	1	2	0	-279.315101	16.02
lin5	Coov	NCH NCH **NC**	0	2	2	0	-279.331218	5.90

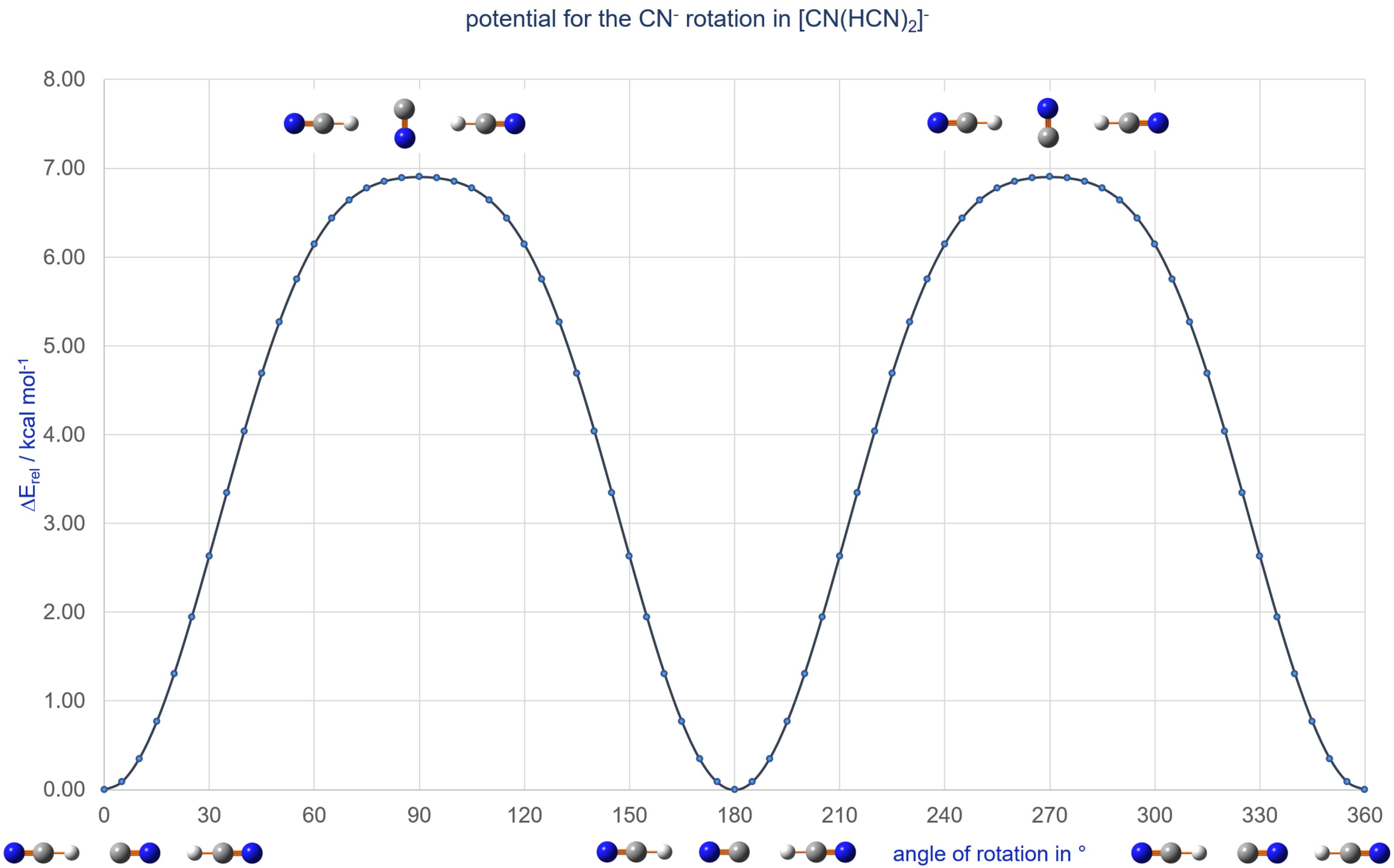

Figure S 14. Potential of rotation (pbe0/aug-cc-pVTZ).

Trisolvate $[CN(HCN)_3]^-$

Table S 8. Absolute energies in a.u., relative energies in kcal/mol at pbe1pbe/aug-cc-pVTZ.

isomer	symmetry	connectivity				number of HN	bonds CH	number of HN_bridge	bonds HC_bridge	E0	H298	G298	ΔE0	ΔH298	ΔG298
i1	Cs	NCH	N C	HCN	HCN	0	3	3	0	-372.891222	-372.820035	-372.877178	4.96	4.81	4.73
i2	Cs	NCH	N C	HCN	HNC	1	2	3	0	-372.876537	-372.805673	-372.861778	14.18	13.82	14.40
i3 opt to I2	Cs	NCH	N to CN C	HNC	HCN	1	2	1	2						
i4 opt to I11	Cs	NCH	N to CN C	HNC	HNC										
i5	Cs	CNH	N C	HCN	HCN	1	2	3	0	-372.878851	-372.807892	-372.864376	12.73	12.43	12.77
i6	Cs	CNH	N C	HCN	HNC	2	1	3	0	-372.863578	-372.792765	-372.848375	22.31	21.92	22.81
i7	Cs	CNH	N C	HNC	HNC	3	0	2	1	-372.852556	-372.783495	-372.838376	29.23	27.74	29.08
i8	Cs	CNH	N C	HNC	HCN	2	1	2	1	-372.866122	-372.796053	-372.851813	20.71	19.86	20.65
l1	Coov	NCH	NCH	CN	HCN	0	3	2	1	-372.896839	-372.825509	-372.879972	1.44	1.37	2.98
l2	Coov	NCH	CNH	NC	HCN	1	2	2	1	-372.888920	-372.820571	-372.869105	6.41	4.47	9.80
l3	Coov	NCH	NCH	NC	HCN	0	3	2	1	-372.896912	-372.825465	-372.879927	1.39	1.40	3.01
l4 opt to l3	Coov	NCH	CNH	CN	HCN	1	2	1	2						
l5	Coov	CNH	NCH	CN	HCN	1	2	2	1	-372.882420	-372.811585	-372.864983	10.49	10.11	12.39
l6	Coov	CNH	NCH	NC	HCN	1	2	2	1	-372.882445	-372.811366	-372.864869	10.47	10.25	12.46

I7	Coov	CNH	CNH	**NC**	**HCN**					-372.877471	-372.808234	-372.859553	13.59	12.22	15.79
I8	Coov	CNH	CNH	**NC**	**HNC**	3	0	1	2	-372.864196	-372.796653	-372.843698	21.92	19.48	25.74
I9	Coov	NCH	NCH	**NC**	**HNC**	1	2	2	1	-372.886652	-372.816418	-372.865397	7.83	7.08	12.13
I10	Coov	NCH	NCH	**CN**	**HNC**	1	2	2	1	-372.885999	-372.815489	-372.864312	8.24	7.66	12.81
I11	Coov	NCH	CNH	**NC**	**HNC**	2	1	1	2	-372.877477	-372.808232	-372.856363	13.59	12.22	17.80
I12	Coov	CNH	NCH	**NC**	**HNC**	2	1	2	1	-372.871916	-372.801888	-372.849937	17.08	16.20	21.83
I13 opt to I12	Coov	CNH	CNH	**CN**	**HNC**										
I14 opt to I6	Coov	NCH	CNH	**CN**	**HNC**										
I15	Coov	CNH	NCH	**CN**	**HNC**	2	1	2	1	-372.871309	-372.801106	-372.848903	17.46	16.69	22.48
y1	C2v	NCH<br>NCH	NC	HCN		0	**3**	2	1	-372.899133	-372.827700	-372.884723	0.00	0.00	0.00
y2	C2v	NCH<br>NCH	CN	HCN		0	**3**	1	2	-372.897043	-372.825517	-372.882216	1.31	1.37	1.57
y3	C2v	NCH<br>NCH	NC	HNC		1	2	2	1	-372.887964	-372.817152	-372.873573	7.01	6.62	7.00
y4	C2v	NCH<br>NCH	CN	HNC		1	2	1	2	-372.885525	-372.814405	-372.870297	8.54	8.34	9.05
y5	C2v	CNH<br>CNH	CN	HNC		3	0	3	0	-372.855751	-372.784928	-372.839471	27.22	26.84	28.40
y6	C2v	CNH<br>CNH	NC	HNC		3	0	2	1	-372.858653	-372.788036	-372.842999	25.40	24.89	26.18
y7	C2v	CNH	NC	HCN		2	1	2	1	-372.870714	-372.799702	-372.855330	17.83	17.57	18.44

		CNH												
y8	C2v	CNH<br><br>CNH	CN	HCN	2	1	1	2	-372.868068	-372.797007	-372.852346	19.49	19.26	20.32
c1	C3v	(NCH)3		NC	0	**3**	3	0	-372.893318	-372.821784	-372.880471	3.65	3.71	2.67
c2	C3v	(NCH)3		CN	0	**3**	0	3	-372.887993	-372.818396	-372.871936	6.99	5.84	8.02
c3	Cs	(NCH)2	CNH	NC	1	2	3	0	-372.880383	-372.809085	-372.867431	11.77	11.68	10.85
c4	Cs	NCH	(CNH)2	NC					-372.864344	-372.793276	-372.851959	21.83	21.60	20.56
c5 opt to y3	Cs	(NCH)2	CNH	CN										
c6 opt to y8	Cs	NCH	(CNH)2	CN										
t1<br>opt to y6	Cs	CNH<br><br>CNH	NC	HNC										
t2	Cs	NCH<br><br>CNH	NC	HNC	2	1	2	1	-372.874642	-372.803898	-372.859462	15.37	14.94	15.85
t3	Cs	CNH<br><br>NCH	NC	HCN	1	2	2	1	-372.886371	-372.815182	-372.871359	8.01	7.86	8.39
t4<br>opt to y3	Cs	NCH<br><br>CNH	CN	HCN										

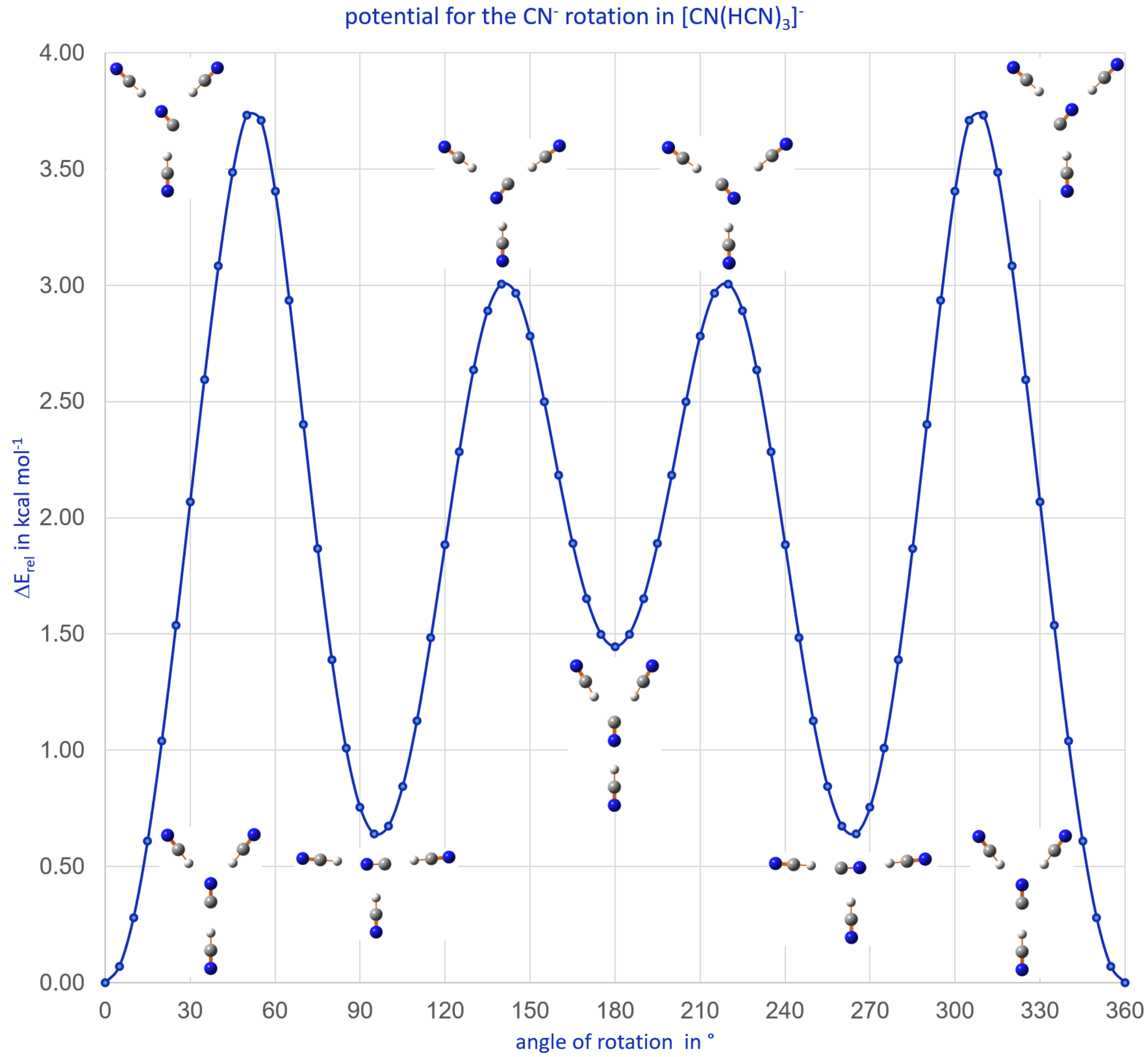

Figure S 15. Potential of rotation (pbe0/aug-cc-pVTZ).

Table S 9. NBO data.

monomers	charges q in e															
**[NCH-CN]$^-$**	N	C	H	C	N										**sumCN$^-$**	**q(CT)**
	-0.46	0.06	0.23	-0.18	-0.65										-0.83	0.17
**[CNH-NC]$^-$**	N	C	H	C	N										**sumCN$^-$**	**q(CT)**
	-0.71	0.07	0.41	0.00	-0.76										-0.76	0.24
**[NCH-NC]$^-$**	**N**	**C**	**H**	**C**	**N**							**sumNCH**	**sumNCH**	**sumNCH**	**sumCN$^-$**	**q(CT)**
**lin1**	**-0.49**	**0.10**	**0.28**	**-0.06**	**-0.83**							**-**	**-**	**-0.11**	**-0.89**	**0.11**
**dimer**																
**[NCH-NCH-CN]$^-$**	**N**	**C**	**H**	**N**	**C**	**H**	**C**	**N**	**H**	**C**	**N**	**sumNCH**	**sumNCH**	**sumNCH**	**sumCN$^-$**	**q(CT)**
**y1**				-0.42	0.09	0.27	**-0.01**	**-0.87**	0.27	0.09	-0.42	-	-0.06	-0.06	-0.88	0.12
**lin1**	**-0.43**	**0.08**	**0.27**	**-0.43**	**0.07**	**0.24**	**-0.07**	**-0.72**				**-**	**-0.08**	**-0.13**	**-0.79**	**0.21**
**trimer**																
**[NCH-CN-(HCN)$_2$]$^-$**	**N**	**C**	**H**	**N**	**C**	**H**	**C**	**N**	**H**	**C**	**N**	**sumNCH**	**sumNCH**	**sumNCH**	**sumCN$^-$**	**q(CT)**
**y1**	**-0.41**	**0.07**	**0.24**	**-0.40**	**0.09**	**0.26**	**-0.02**	**-0.78**	**0.26**	**0.09**	**-0.40**	**-0.10**	**-0.05**	**-0.05**	**-0.80**	**0.20**
**lin1**	**-0.38**	**0.08**	**0.26**	**-0.50**	**0.13**	**0.23**	**-0.06**	**-0.69**	**0.27**	**0.08**	**-0.42**	**-0.04**	**-0.14**	**-0.07**	**-0.75**	**0.25**

Table S10. Reaction energies (pbe0/aug-cc-pVTZ) for the stepwise formation of solvates in kcal/mol (for the thermodynamically most favored isomer).

**[NC-H-NC]$^-$**	**Coov**	**HCN + CN$^-$ → monomer$^-$**		
**ΔE0**		-23.40		
**ΔH298**		-23.59		
**ΔG298**		-15.91		
**dimer lin1**	**Coov**	**2 HCN + CN$^-$ → dimer$^-$**	**HCN + monomer$^-$ → dimer$^-$**	
**ΔE0**		-42.06	-18.66	
**ΔH298**		-42.28	-18.69	
**ΔG298**		-26.97	-11.06	
**trimer y1**	**C2v**	**3 HCN + CN$^-$ → trimer$^-$**	**2 HCN + monomer$^-$ → trimer$^-$**	**HCN + dimer$^-$ → trimer$^-$**
**ΔE0**		-55.03	-31.63	-12.97
**ΔH298**		-55.25	-31.65	-12.96
**ΔG298**		-34.02	-18.11	-7.05

Table S11. Reaction energies (pbe0/aug-cc-pVTZ, pcm(MeCN)) for the stepwise formation of solvates in kcal/mol (for the thermodynamically most favored isomer).

**[NC-H-NC]$^-$**	**Coov**	**HCN + CN$^-$ → monomer$^-$**		
**ΔE0**		-6.27		
**ΔH298**		-6.37		
**ΔG298**		1.02		
**dimer lin1**	**Coov**	**2 HCN + CN$^-$ → dimer$^-$**	**HCN + monomer$^-$ → dimer$^-$**	
**ΔE0**		-11.91	-5.64	
**ΔH298**		-11.90	-5.56	
**ΔG298**		2.37	1.35	
**trimer y1**	**C2v**	**3 HCN + CN$^-$ → trimer$^-$**	**2 HCN + monomer$^-$ → trimer$^-$**	**HCN + dimer$^-$ → trimer$^-$**
**ΔE0**		-15.93	-9.66	-4.02
**ΔH298**		-15.91	-9.57	-4.01
**ΔG298**		4.58	3.56	2.21

Table S12. Reaction energies (pbe0/a3ug-cc-pVTZ, pcm(CH_2Cl_2)) for the stepwise formation of solvates in kcal/mol (for the thermodynamically most favored isomer).

$[NC\text{-}H\text{-}NC]^-$	Coov	$HCN + CN^- \rightarrow$ monomer$^-$		
ΔE0		-7.85		
ΔH298		-7.95		
ΔG298		-0.44		
dimer lin1	Coov	$2\ HCN + CN^- \rightarrow$ dimer$^-$	HCN + monomer$^- \rightarrow$ dimer$^-$	
ΔE0		-14.80	-6.96	
ΔH298		-14.83	-6.89	
ΔG298		-0.42	0.02	
trimer y1	C2v	$3\ HCN + CN^- \rightarrow$ trimer$^-$	$2\ HCN$ + monomer$^- \rightarrow$ trimer$^-$	HCN + dimer$^- \rightarrow$ trimer$^-$
ΔE0		-19.79	-11.94	-4.98
ΔH298		-19.78	-11.83	-4.94
ΔG298		0.41	0.85	0.83

Table S13. Reaction energies (pbe0/aug-cc-pVTZ, smd(MeCN)) for the stepwise formation of solvates in kcal/mol (for the thermodynamically most favored isomer).

$[NC\text{-}H\text{-}NC]^-$	Coov	$HCN + CN^- \rightarrow$ monomer$^-$		
ΔE0		-3.79		
ΔH298		-3.73		
ΔG298		2.87		
dimer lin1	Coov	$2\ HCN + CN^- \rightarrow$ dimer$^-$	HCN + monomer$^- \rightarrow$ dimer$^-$	
ΔE0		-6.49	-2.71	
ΔH298		-6.36	-2.64	
ΔG298		7.61	4.73	
trimer y1	C2v	$3\ HCN + CN^- \rightarrow$ trimer$^-$	$2\ HCN$ + monomer$^- \rightarrow$ trimer$^-$	HCN + dimer$^- \rightarrow$ trimer$^-$
ΔE0		-9.17	-5.38	-2.67
ΔH298		-8.99	-5.26	-2.63
ΔG298		11.24	8.37	3.63

Figure S 16. Selected computed structures (best isomer of each class, pbe0/aug-cc-pVTZ).

Monomer

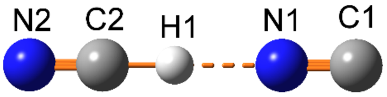

L1

Dimer

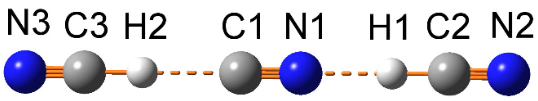

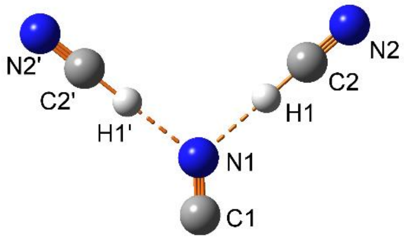

L_D1

Y_D1

Trimer

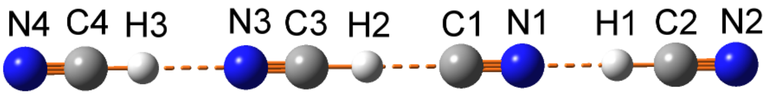

L_T1

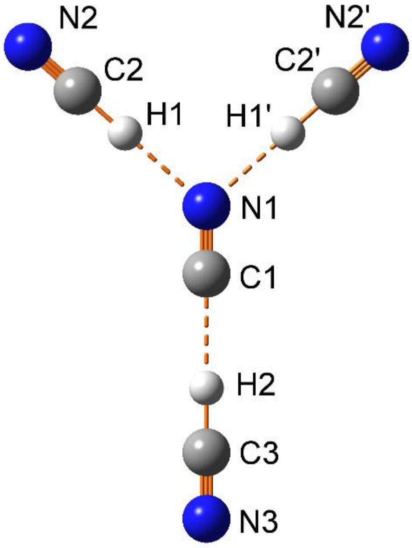

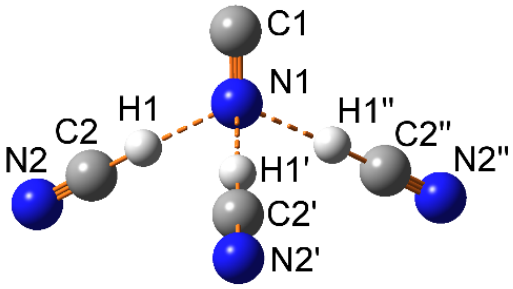

Y_T1

C1

Table S14. Selected structural data, distances in Å, angles in ° (pbe0/aug-cc-pvtz, see Figure S16 for numbering of atoms)

	monomer	*dimer*			*trimer*			unit1	unit 2
**distances**	**L1**	L_D1	$\mathbf{Y_D1}$	exp[a]	$\mathbf{L_T1}$	$\mathbf{Y_T1}$	**C1**	exp[b,e,f]	[e,f]
C1-N1	1.169	1.165	1.171	1.032(5)[h]	1.163	1.166	1.173	1.133(3)	1.130(3)
N1-H1	1.693	1.774	1.838	1.900	1.802	1.891	1.942	2.144/2.163(2)	2.147/2.158[g]
H1-C2	1.139	1.119	1.107	1.05	1.113	1.098	1.095	0.95/0.95[g]	0.95/0.95[g]
C2-N2	1.151	1.150	1.149	1.087(9)[h]	1.149	1.148	1.148	1.111(3)/1.118(3)	1.108(3)/1.117(2)[g]
C1-H2	-	1.918	-[c]	2.27[i]	1.789	1.981	-[d]	2.197	2.205[g]
H2-C3	-	1.125	-[c]	1.05[i]	1.155	1.112	-[d]	0.95[g]	0.95[g]
C3-N3	-	1.150	-[c]	1.087(9)[h]	1.149	1.149	-[d]	1.124(3)	1.122(3)
N3-H3	-	-	-	-	1.958	-[c]	-[d]	-	-
H3-C4	-	-	-	-	1.090	-[c]	-[d]	-	-
C4-N4	-	-	-	-	1.148	-[c]	-[d]	-	-
**angles**									
C1-N1-H1	180.0	180.0	129.6	169.8[i]	180.0	131.9	115.8	135.1/126.1	135.0/125.8[f]
N1-H1-C2	180.0	180.0	179.8	173.5[i]	180.0	179.9	177.9	162.6/169.0	161.7/167.8[f]
H1-C2-N2	180.0	180.0	180.0	173.5[i]	180.0	180.0	179.9	180.0/180.0	180.0/180.0[f]
N1-C1-H2	-	180.0	-[c]		180.0	180.0	-	157.6	157.9[f]
C1-H2-C3	-	180.0	-[c]		180.0	180.0	-	171.7	172.1[f]
H2-C3-N3	-	180.0	-[c]		180.0	180.0	-	180.0	180.0[f]
C3-N3-H3	-	-	-		180.0	-	-	-	-
N3-H3-C4	-	-	-		180.0	-	-	-	-
H3-C4-N4	-	-	-		180.0	-	-	-	-
**dihedral angles**									
C1-N1-C2-C2'	-	-	180.0		-	180.0	127.8	132.3	132.7

[a] closely related to computed gas phase isomer $\mathbf{L_D1}$ (Note: No cation/anion and anion/anion interaction considered in the gas phase calculation! Hence, a comparison should be done very carefully); $\mathbf{L_D1}$ is linear but exp-structure slightly bent
[b] closely related to computed gas phase isomer $\mathbf{Y_T1}$ (Note: No cation/anion and anion/anion interaction considered in the gas phase calculation! Hence, a comparison should be done very carefully) $\mathbf{Y_T1}$ is planar but exp-structure strongly distorted from planarity
[c] Due to C2v symmetry equivalent to H1-C2-N2
[d] Due to C3v symmetry equivalent to H-C2-N2
[e] Due independent anions
[f] In contrast to the related YT1 isomer these anions are non-planar and only C1 symmetric
[g] calculated at 0.95 Å, therefore no SD for any H parameters
[f] No SD given since H atom position was computed at 0.95
[h] to short due to disorder
[i] calculated at 1.05, therefore no SD for any H parameters

Table S15. Selected structural data, distances in Å, angles in ° (pbe0/aug-cc-pvtz, pcm(MeCN), see Figure S16 for numbering of atoms)

	monomer	*dimer*			*trimer*			unit1	unit 2
**distances**	**L1**	**L_D1**	**Y_D1**	exp[a]	**L_T1**	**Y_T1**	**C1**	exp[b,e,f]	[e,f]
C1-N1	1.168	1.165	1.169	1.032(5)[h]	1.165	1.166	1.169	1.133(3)	1.130(3)
N1-H1	1.823	1.845	1.908	1.900	1.849	1.927	1.967	2.144/2.163(2)	2.147/2.158[g]
H1-C2	1.108	1.105	1.096	1.05	1.104	1.093	1.090	0.95/0.95[g]	0.95/0.95[g]
C2-N2	1.148	1.148	1.147	1.087(9)[h]	1.148	1.147	1.147	1.111(3)/1.118(3)	1.108(3)/1.117(2)[g]
C1-H2	-	1.991	-[c]	2.27[i]	1.946	2.012	-[d]	2.197	2.205[g]
H2-C3	-	1.109	-[c]	1.05[i]	1.116	1.105	-[d]	0.95[g]	0.95[g]
C3-N3	-	1.148	-[c]	1.087(9)[h]	1.147	1.148	-[d]	1.124(3)	1.122(3)
N3-H3	-	-	-	-	2.018	-[c]	-[d]	-	-
H3-C4	-	-	-	-	1.085	-[c]	-[d]	-	-
C4-N4	-	-	-	-	1.147	-[c]	-[d]	-	-
**angles**									
C1-N1-H1	180.0	180.0	136.1	169.8[i]	180.0	137.6	127.3	135.1/126.1	135.0/125.8[f]
N1-H1-C2	180.0	180.0	179.6	173.5[i]	180.0	179.4	178.1	162.6/169.0	161.7/167.8[f]
H1-C2-N2	180.0	180.0	180.0	173.5[i]	180.0	179.9	179.9	180.0/180.0	180.0/180.0[f]
N1-C1-H2	-	180.0	-[c]		180.0	179.7	-	157.6	157.9[f]
C1-H2-C3	-	180.0	-[c]		180.0	179.7	-	171.7	172.1[f]
H2-C3-N3	-	180.0	-[c]		180.0	179.9	-	180.0	180.0[f]
C3-N3-H3	-	-	-		180.0	-	-	-	-
N3-H3-C4	-	-	-		180.0	-	-	-	-
H3-C4-N4	-	-	-		180.0	-	-	-	-
**dihedral angles**									
C1-N1-C2-C2’	-	-	180.0		-	180.0	137.8	132.3	132.7

[a] closely related to computed gas phase isomer **L_D1** (Note: No cation/anion and anion/anion interaction considered in the gas phase calculation! Hence, a comparison should be done very carefully); **L_D1** is linear but exp-structure slightly bent
[b] closely related to computed gas phase isomer **Y_T1** (Note: No cation/anion and anion/anion interaction considered in the gas phase calculation! Hence, a comparison should be done very carefully) **Y_T1** is planar but exp-structure strongly distorted from planarity
[c] Due to C2v symmetry equivalent to H1-C2-N2
[d] Due to C3v symmetry equivalent to H-C2-N2
[e] Due independent anions
[f] In contrast to the related YT1 isomer these anions are non-planar and only C1 symmetric
[g] calculated at 0.95 Å, therefore no SD for any H parameters
[f] No SD given since H atom position was computed at 0.95
[h] to short due to disorder
[i] calculated at 1.05, therefore no SD for any H parameters

Table S16. Selected structural data, distances in Å, angles in ° (pbe0/aug-cc-pvtz, pcm(CH_2Cl_2), see Figure S16 for numbering of atoms)

distances	*monomer* L1	*dimer* L_D1	Y_D1	exp[a]	*trimer* L_T1	Y_T1	C1	unit1 exp[b,e,f]	unit 2 [e,f]
C1-N1	1.168	1.165	1.169	1.032(5)[h]	1.165	1.166	1.170	1.133(3)	1.130(3)
N1-H1	1.807	1.835	1.894	1.900	1.842	1.920	1.959	2.144/2.163(2)	2.147/2.158[g]
H1-C2	1.111	1.106	1.097	1.05	1.105	1.094	1.091	0.95/0.95[g]	0.95/0.95[g]
C2-N2	1.149	1.148	1.148	1.087(9)[h]	1.148	1.147	1.147	1.111(3)/1.118(3)	1.108(3)/1.117(2)[g]
C1-H2	-	1.982	-[c]	2.27[i]	1.928	2.008	-[d]	2.197	2.205[g]
H2-C3	-	1.110	-[c]	1.05[i]	1.119	1.106	-[d]	0.95[g]	0.95[g]
C3-N3	-	1.149	-[c]	1.087(9)[h]	1.147	1.148	-[d]	1.124(3)	1.122(3)
N3-H3	-	-	-	-	2.012	-[c]	-[d]	-	-
H3-C4	-	-	-	-	1.085	-[c]	-[d]	-	-
C4-N4	-	-	-	-	1.147	-[c]	-[d]	-	-
**angles**									
C1-N1-H1	180.0	180.0	138.0	169.8[i]	180.0	137.2	126.0	135.1/126.1	135.0/125.8[f]
N1-H1-C2	180.0	180.0	179.0	173.5[i]	180.0	179.6	177.9	162.6/169.0	161.7/167.8[f]
H1-C2-N2	180.0	180.0	180.0	173.5[i]	180.0	179.9	179.9	180.0/180.0	180.0/180.0[f]
N1-C1-H2	-	180.0	-[c]		180.0	179.7	-	157.6	157.9[f]
C1-H2-C3	-	180.0	-[c]		180.0	179.9	-	171.7	172.1[f]
H2-C3-N3	-	180.0	-[c]		180.0	180.0	-	180.0	180.0[f]
C3-N3-H3	-	-	-		180.0	-	-	-	-
N3-H3-C4	-	-	-		180.0	-	-	-	-
H3-C4-N4	-	-	-		180.0	-	-	-	-
**dihedral angles**									
C1-N1-C2-C2'	-	-	180.0		-	180.0	136.2	132.3	132.7

[a] closely related to computed gas phase isomer **L_D1** (Note: No cation/anion and anion/anion interaction considered in the gas phase calculation! Hence, a comparison should be done very carefully); **L_D1** is linear but exp-structure slightly bent
[b] closely related to computed gas phase isomer **Y_T1** (Note: No cation/anion and anion/anion interaction considered in the gas phase calculation! Hence, a comparison should be done very carefully) **Y_T1** is planar but exp-structure strongly distorted from planarity
[c] Due to C2v symmetry equivalent to H1-C2-N2
[d] Due to C3v symmetry equivalent to H-C2-N2
[e] Due independent anions
[f] In contrast to the related YT1 isomer these anions are non-planar and only C1 symmetric
[g] calculated at 0.95 Å, therefore no SD for any H parameters
[f] No SD given since H atom position was computed at 0.95
[h] to short due to disorder
[i] calculated at 1.05, therefore no SD for any H parameters

Table S17. Selected structural data, distances in Å, angles in ° (pbe0/aug-cc-pvtz, smd(MeCN), see Figure S16 for numbering of atoms)

	monomer	*dimer*			*trimer*			unit1	unit 2
**distances**	**L1**	**L_D1**	**Y_D1**	exp[a]	**L_T1**	**Y_T1**	**C1**	exp[b,e,f]	[e,f]
C1-N1	1.166	1.165	1.167	1.032(5)[h]	1.165	1.165	1.167	1.133(3)	1.130(3)
N1-H1	1.874	1.887	1.967	1.900	1.889	1.970	2.030	2.144/2.163(2)	2.147/2.158[g]
H1-C2	1.102	1.101	1.092	1.05	1.101	1.091	1.088	0.95/0.95[g]	0.95/0.95[g]
C2-N2	1.147	1.147	1.147	1.087(9)[h]	1.147	1.146	1.147	1.111(3)/1.118(3)	1.108(3)/1.117(2)[g]
C1-H2	-	2.057	-[c]	2.27[i]	2.028	2.077	-[d]	2.197	2.205[g]
H2-C3	-	1.102	-[c]	1.05[i]	1.106	1099	-[d]	0.95[g]	0.95[g]
C3-N3	-	1.147	-[c]	1.087(9)[h]	1.146	1.147	-[d]	1.124(3)	1.122(3)
N3-H3	-	-	-	-	2.076	-[c]	-[d]	-	-
H3-C4	-	-	-	-	1.084	-[c]	-[d]	-	-
C4-N4	-	-	-	-	1.146	-[c]	-[d]	-	-
**angles**									
C1-N1-H1	180.0	180.0	142.5	169.8[i]	180.0	142.4	135.7	135.1/126.1	135.0/125.8[f]
N1-H1-C2	180.0	180.0	177.4	173.5[i]	180.0	177.3	172.5	162.6/169.0	161.7/167.8[f]
H1-C2-N2	180.0	180.0	179.9	173.5[i]	180.0	179.9	179.6	180.0/180.0	180.0/180.0[f]
N1-C1-H2	-	180.0	-[c]		180.0	179.3	-	157.6	157.9[f]
C1-H2-C3	-	180.0	-[c]		180.0	179.8	-	171.7	172.1[f]
H2-C3-N3	-	180.0	-[c]		180.0	180.0	-	180.0	180.0[f]
C3-N3-H3	-	-	-		180.0	-	-	-	-
N3-H3-C4	-	-	-		180.0	-	-	-	-
H3-C4-N4	-	-	-		180.0	-	-	-	-
**dihedral angles**									
C1-N1-C2-C2'	-	-	180.0		-	180.0	144.8	132.3	132.7

[a] closely related to computed gas phase isomer **L_D1** (Note: No cation/anion and anion/anion interaction considered in the gas phase calculation! Hence, a comparison should be done very carefully); **L_D1** is linear but exp-structure slightly bent
[b] closely related to computed gas phase isomer **Y_T1** (Note: No cation/anion and anion/anion interaction considered in the gas phase calculation! Hence, a comparison should be done very carefully) **Y_T1** is planar but exp-structure strongly distorted from planarity
[c] Due to C2v symmetry equivalent to H1-C2-N2
[d] Due to C3v symmetry equivalent to H-C2-N2
[e] Due independent anions
[f] In contrast to the related YT1 isomer these anions are non-planar and only C1 symmetric
[g] calculated at 0.95 Å, therefore no SD for any H parameters
[f] No SD given since H atom position was computed at 0.95
[h] to short due to disorder
[i] calculated at 1.05, therefore no SD for any H parameters

5. References

[1] L. Cattelan, M. Noè, M. Selva, N. Demitri, A. Perosa, *ChemSusChem* **2015**, *8*, 3963–3966.

[2] K. Bläsing, J. Bresien, R. Labbow, A. Schulz, A. Villinger, *Angew. Chem. Int. Ed.* **2018**, *57*, 9170–9175.

[3] G. R. Fulmer, A. J. M. Miller, N. H. Sherden, H. E. Gottlieb, A. Nudelman, B. M. Stoltz, J. E. Bercaw, K. I. Goldberg, *Organometallics* **2010**, *29*, 2176–2179.

[4] J. Harloff, A. Schulz, P. Stoer, A. Villinger, *Z. Anorg. Allg. Chem.* **2019**, *645*, 835–839.

[5] J. Harloff, D. Michalik, S. Nier, A. Schulz, P. Stoer, A. Villinger, *Angew. Chem. Int. Ed.* **2019**, *58*, 5452–5456.

[6] S. Arlt, J. Harloff, A. Schulz, A. Stoffers, A. Villinger, *Inorg. Chem.* **2016**, *55*, 12321–12328.

[7] Z. M. Smallwood, M. F. Davis, J. G. Hill, L. J. R. James, P. Portius, *Inorg. Chem.* **2019**, *58*, 4583–4591.

[8] J. Bresien, C. Hering-Junghans, P. Kumm, A. Schulz, M. Thomas, A. Villinger, *Eur. J. Inorg. Chem.* **2018**, *2018*, 647–651.

[9] *Gaussian 09, Revision C.01,* M. J. Frisch, G. W. Trucks, H. B. Schlegel, G. E. Scuseria, M. A. Robb, J. R. Cheeseman, G. Scalmani, V. Barone, B. Mennucci, G. A. Peterson, H. Nakatsuji, M. Caricato, X. Li, H. P. Hratchian, A. F. Izmaylov, J. Bloino, G. Zheng, J. L. Sonnenberg, M. Hada, M. Ehara, K. Toyota, R. Fukuda, J. Hasegawa, M. Ishida, T. Nakajima, Y. Honda, O. Kitao, H. Nakai, T. Vreven, J. A. Montgomery Jr., J. E. Peralta, F. Ogliaro, M. Bearpark, J. J. Heyd, E. Brothers, K. N. Kudin, V.N. Staroverov, T. Keith, R. Kobayashi, J. Normand, K. Raghavachari, A. Rendell, J. C. Burant, S. S. Iyengar, J. Tomasi, M. Cossi, N. Rega, J. M. Millam, M. Klene, J. E. Know, J. B. Cross, V. Bakken, C. Adamo, J. Jaramillo, R. Gomperts, R. E. Stratmann, O.Yazyev, A. J. Austin, R. Cammi, C. Pomelli, J. W. Ochterski, R. L. Martin, K. Morokuma, V. G. Zakrezewski, G. A. Voth, P. Salvador, J. J. Dannenberg, S. Dapprich, A. D. Daniels, O. Farkas, J. B. Foresman, J. V Ortiz, J. Cioslowski and D. J. Fox, Gaussian, Inc., Wallingford CT, **2010**.

[10] F. Weinhold, C. R. Landis, E. D. Glendening, *Int. Rev. Phys. Chem.* **2016**, *35*, 399–440.

[11] E. D. Glendening, F. Weinhold, *J. Comput. Chem.* **1997**, *19*, 610–627.

[12] E. D. Glendening, F. Weinhold, *J. Comput. Chem.* **1998**, *19*, 593–609.

[13] E. D. Glendening, C. R. Landis, F. Weinhold, *J. Comput. Chem.* **2013**, *34*, 1429–1437.

[14] S. A. Chacko, I. H. Krouse, L. A. Hammad, P. G. Wenthold, *J. Am. Soc. Mass Spectrom.* **2006**, *17*, 51–55.

[15] P. Kollman, J. McKelvey, A. Johansson, S. Rothenberg, *J. Am. Chem. Soc.* **1975**, *97*, 955–965.

[16] M. Meot-Ner, *J. Am. Chem. Soc.* **1978**, *100*, 4694–4699.

[17] J. W. Larson, T. B. Mcmahon, *Inorg. Chem.* **1984**, *23*, 2029–2033.

[18] M. Meot-Ner, S. M. Cybulski, S. Scheiner, J. F. Liebman, *J. Phys. Chem.* **1988**, *92*, 2738–2745.

[19] M. Sánchez, P. F. Provasi, G. A. Aucar, I. Alkorta, J. Elguero, *J. Phys. Chem. B* **2005**, *109*, 18189–18194.
